# Simplified Method of Multi-Elemental Analysis of Dialyzable Fraction of Tea Infusions by FAAS and ICP OES

**DOI:** 10.1007/s12011-019-01828-x

**Published:** 2019-07-24

**Authors:** Anna Szymczycha-Madeja, Maja Welna, Pawel Pohl

**Affiliations:** Department of Analytical Chemistry and Chemical Metallurgy, Faculty of Chemistry, Wroclaw University of Technology, Smoluchowskiego 23,, 50-372 Wroclaw, Poland

**Keywords:** Tea infusions, In vitro gastrointestinal digestion, Mass balance study, Method validation, Spectrometric methods, statistical analysis

## Abstract

A fast and straightforward sample preparation procedure of the dialyzable fraction of infusions of teas prior to their analysis on Al, Ba, Ca, Cu, Fe, Mg, Mn, Ni, Sr, and Zn contents by flame atomic absorption spectrometry (FAAS) and inductively coupled plasma optical emission spectrometry (ICP OES) was developed and validated. The proposed methodology was based on acidification with HNO_3_ only and demonstrated good analytical performance, i.e., precision (0.80–5.0%), accuracy (< 5%), recoveries of elements (97.4–105%), and their detection limits (0.075–1.1 μg L^−1^) along with linearity of calibration curves in the whole studied concentration ranges. Applicability of the evaluated procedure, being a useful alternative to time-consuming wet digestions, was tested by determining bioaccessibility of elements in 20 infusions of black (BT) and green (GT) teas as assessed with the aid of in vitro gastrointestinal digestion. Average contributions of bioaccessible fractions (%) of studied metals were as follows: 1.18 (Al)–40.7% (Ca) and 4.65% (Al)–46.3% (Ca) for BTs and GTs, respectively. Drinking daily four cups (1 L) of tea, recommended dietary intakes (RDIs) of Ca, Cu, Fe, Mg, and Zn were covered to a small degree (< 1.5%). Only bioaccessibility of Mn highly contributed to RDI for this metal. According to provisional tolerable weekly intakes (PTWIs) for toxic elements such as Al and Ni, consumption of both types of teas should not represent any health risk. Additionally, analysis of variance of results clearly indicated that BTs and GTs were mostly differentiated due to concentrations of the bioaccessible fraction of Al, Ba, Cu, and Ni.

## Introduction

Brewed teas are a valuable source of antioxidants as well as essential elements. Element analysis of tea infusions is overwhelmingly concerned on determination of total concentrations of various metals to assess quality and safety of this ubiquitous beverage or estimate recommended daily intakes (RDIs) for nutritionally relevant or toxic metals. In the latter case, it was always assumed that metals released into infusions are 100% bioaccessible [1]. Actually, to understand nutritional benefits or possible harmful implications of tea infusions to human health, bioaccessibility of metals using in vitro digestion with artificial enzymes should be evaluated, mimicking processes in the gastrointestinal tract. Traditionally, a two-step procedure with solutions of pepsin and mixtures of pancreatin and bile salts are commonly used to simulate gastrointestinal digestion (GID). Typically, to reflect body temperature and digestion duration, GID has been proceeded at 37 °C within 2 h for each digestion step (associated with gentle shaking to simulate gastric and intestinal peristalsis). Gastric digestion is usually performed using solutions of pepsin (0.001–16%, pH ~ 2), while for intestinal digestion, solutions of pancreatin (0.015–3.04%) and bile salts (0.15–2.8%) adjusted to pH ~ 7 by 0.1 or 1.0 mol L^−1^ Na_2_CO_3_ [2, 3] are mostly applied. Normally, digested samples are separated from solutions by centrifugation and the soluble fraction of metals is assessed in resultant supernatants (bioavailability). To imitate absorption of metals in the villi, dialysis with semipermeable membranes can be additionally added, enabling to assess the dialyzable fraction of metals (bioaccessibility). Concentrations of metals in both separated soluble or dialyzable fractions are determined by spectrometric methods such as flame atomic absorption spectrometry (FAAS), inductively coupled plasma optical emission spectrometry (ICP OES), or inductively coupled plasma mass spectrometry (ICP-MS) [4–25].

Bioaccessibility of different metals has been estimated so far in breads [4], fruits and vegetables [4–7], fruit juices [8, 9], coffees [10–12], milks [13, 14], herbal remedies [15], infant formulas [13, 16], meat [17], linseed, sesame and cereals [16, 18], and edible seaweeds [19–21]. Unfortunately, just few papers were devoted so far to bioavailability/bioaccessibility of selected metals from tea infusions. Accordingly, Powell et al. [22] assessed bioaccessibility of Al, Ca, Cu, Fe, K, Mg, Mn, Na, and Zn from one BT infusions. Samples of infusions were incubated with gastric juice and then adjusted to pH 6.5 to simulate intestinal pH. Next, they simulated absorption of metals in the villi by ultrafiltration over membranes with 3, 10, and 30 kDa molecular weight cut-offs (MWCOs). Resulted sample solutions were diluted (1:1), acidified with HNO_3_ to 0.11 mol L^−1^, and measured by ICP OES. Lin and Yang [23] determined bioavailability of Al from infusions of three teas (GT, BT, and oolong tea), but without membrane ultrafiltration. Samples of infusions after in vitro GID were centrifuged, and their supernatants were analyzed by FAAS. Similarly, contributions of the bioavailable fraction of Li [24] and Mg, Mn, and Fe [25] from infusions of six BTs and GTs were evaluated. As before, no membrane ultrafiltration was used to imitate absorption of these metals in the villi. After in vitro GID, samples were just centrifuged, filtered, and measured directly using ICP-MS.

Unfortunately, there is no standardized and fully validated procedure for preparing sample solutions after in vitro GID prior to their multi-element analysis by spectrometric methods. Development of simplified sample preparation procedures of the dialyzable fraction of tea infusions before such analysis and evaluation of bioaccessibility seems to be of a special significance because it is a critical step of the whole analytical chain. Its application is important in reference to obtain reliable results on bioaccessibility of metals in brewed teas and their nutritional value, which actually does not depend on the total content of metals but absorption/assimilation in the gastrointestinal tract.

Hence, the first aim of this work was to develop and validate a simple and fast, non-digestion sample preparation procedure useful for assessing the dialyzable fraction of elements in infusions of black (BT) and green (GT) teas by means of FAAS (Ca and Mg) and ICP OES (Al, Ba, Cu, Fe, Mn, Ni, Sr, and Zn). Suitability of no sample treatment (direct analysis) and acidification with HNO_3_, both alternatively used to wet digestion, was evaluated in terms of selected figures of merit, i.e., precision and accuracy of results, and limits of detection (LODs) of metals. This is the first report on methodical comparison of simplified sample preparation procedures used for evaluating the dialyzable fraction of metals (Al, Ba, Ca, Cu, Fe, Mg, Mn, Ni, Sr, and Zn) in in vitro gastrointestinal digested infusions of BTs and GTs. Due to the popularity of tea infusions worldwide and limited knowledge about their nutritional value or possible harmful effects on human health, the second aim of this work was to evaluate the bioaccessibility of Al, Ba, Ca, Cu, Fe, Mg, Mn, Ni, Sr, and Zn from infusions of bagged and leafy black (BTBs and BTLs) and green (GTBs and GTLs) teas after application of in vitro GID by using the proposed preparation procedure. To our best knowledge, this is the first report on determination of bioaccessibility of 10 metals from 20 infusions of BTs and GTs after in vitro simulating GID with absorption of metals species in the villi by using dialysis membranes. Moreover, total concentrations of metals in infusions of BTBs, BTLs, GTBs, and GTLs and their concentrations in dialyzable and non-dialyzable fractions separated from these infusions were applied to differentiate and classify all analyzed teas by means of two-side one-way analysis of variance (ANOVA) and linear discriminant analysis (LDA).

## Experimental

### Samples and Reagents

The most popular and commercially available in Poland BTs and GTs were analyzed. Teas sold in bags and their equivalents in the form of loose leaves were selected (20 in total), i.e., 5 BTBs, 5 GTBs, 5 BTLs, and 5 GTLs.

Merck (Germany) ACS reagents, i.e., concentrated HNO_3_ (65%, *m*/*m*) and HCl (37%, *m*/*m*), pepsin from porcine gastric mucosa (800–2500 units/mg of protein), pancreatin from porcine pancreas, bile salts, PIPES ((piperazine-NN-bis(2-ethane-sulfonic acid) disodium salt)), NaCl, and NaHCO_3_, were used. Freshly prepared solutions of simulated gastric (SGJ) and intestinal (SIJ) juices were applied for GID. They contained 0.32% (*m*/*v*) pepsin with 0.20% (*m*/*v*) NaCl in 0.08 mol L^−1^ HCl (SGJ) and 0.40% (*m*/*v*) pancreatin with 2.5% (*m*/*v*) bile salts in 0.10 mol L^−1^ NaHCO_3_ (SIJ). De-ionized water was used throughout. A Merck Certipur® multi-elemental stock (1000 mg L^−1^) ICP standard solution IV was used to prepare simple and matrix-matched standard solutions for calibration of FAAS and ICP OES.

A high-retention cellulose dialysis tubing of 12.4 kDa MWCO (Sigma-Aldrich, Germany) was used to separate the bioaccessible fraction of studied metals from incubates of infusions of BTs and GTs.

### Instrumentation

Concentrations of Ca and Mg were measured by a Perkin-Elmer single-beam flame atomic absorption spectrophotometer (FAAS), model 1100B. Operating settings recommended by the instrument manufacturer were applied, i.e., lines 422.7 (Ca) and 285.2 nm (Mg), spectral band-passes: 0.7 nm, gas flow rates 8.0 (air) and 1.5 L min^−1^ (fuel), and lamp current 15 mA. Averaged readings of background-corrected absorbances (3 replicates, *n* = 3), taken within 3.0 s in a time-average integration mode, were used for calibration. Working standard solutions for five-point calibration curves were within 0.1–5.0 μg mL^−1^.

The remaining metals (Al, Ba, Cu, Fe, Mn, Ni, Sr, and Zn) were determined using an Agilent ICP OES instrument, model 720. It was operated under typical settings, i.e., the RF power 1.2 kW; gas flow rates 15.0 (plasma), 1.5 (auxiliary), and 0.75 L min^−1^ (nebulizer); sample flow rate 0.75 mL min^−1^; stabilization and sample uptake delays 15 and 30 s; rinse and replicate times 10 and 1 s, respectively; and number of replicates 3. Analytical lines were as follows: Al 396.2, Ba 455.4, Cu 324.8, Fe 259.9, Mn 257.6, Ni 231.6, Sr 407.8, and Zn 213.8 nm. Mean background-corrected intensities of these lines were used for calibration. Working standard solutions used for five-point calibration curves were within 0.1–5.0 μg mL^−1^.

## Sample Preparation Prior to Analysis

### Infusion

Considering steeping time and water temperature, infusions of BTs and GTs were prepared according to recommendations given by tea producers/suppliers. For BTs, contents of bags (2.0 g) or portions of leaves (2.0 g) were placed in 400-mL glass beakers, poured with 200 mL of boiling de-ionized water and left under the cover to infuse for 5 min. Then, infusions were separated from settled grounds by filtering them through 390 grade quantitative filter papers (Munktell & Filtrak, Germany). For GTs, the same masses of samples (2.0 g) were taken, poured with 200 mL of hot de-ionized water (85 °C), and infused for 3 min under the cover. Collected filtrates were split out and then one part was analyzed by FAAS and ICP OES on total concentrations of metals, while another part was subjected to in vitro GID.

### In Vitro GID Procedure

Composition of SGJs and SIJs was selected based on protocols reported for various food products [8, 13, 14, 20, 21]. Because tea is consumed as a fluid and rapidly passes from an oral cavity to a stomach, a mechanical process of chewing was ignored. A PIPES buffer solution was used instead of a NaHCO_3_ solution to obtain physiological pH value (7.0). Its buffering capacity was independent of temperature and concentrations of samples components [9, 19].

For in vitro GID, aliquots of BTs and GTs infusions (20 g) were placed in 50-mL PP tubs, adjusted to pH 2.0 with a HCl solution (6.0 mol L^−1^), and filled with 3.0 mL of a SGJ solution to simulate gastric digestion. Samples were incubated (a temperature-controlled shaking water bath was used) at 37 °C with agitation (150 rpm) for 2 h and then enzymatic reaction was stopped by placing tubes for 10 min into an ice-bath. After this, 5.0 mL of a SIJ solution was added to simulate intestinal digestion. Dialysis membrane tubings with 20 mL of a PIPES solution (0.15 mol L^−1^, pH 7.5 adjusted with HCl) were placed inside these tubes and incubation was continued (37 °C, agitation 150 rpm) for the next 2 h. Then, enzymatic reaction was stopped again (ice-bath, 10 min). Next, contents of dialysis membrane tubings (dialyzable or bioaccessible fraction) and residual solutions of tubes (non-dialyzable fraction, or residue) were transferred to 30-mL PP containers.

### Sample Treatment and Analysis

For preparation of dialyzable and non-dialyzable fractions of infusions of BTs and GTs prior to analysis, preliminary, three different procedures (P1-P3) were used and their reliability was compared. It included wet digestion (P1), direct analysis without any initial treatment (P2), and acidification with HNO_3_ (P3). Wet digestion (P1) was taken as the reference procedure, i.e., giving reference concentrations of metals after subjection to FAAS and ICP OES measurements. For wet digestion (P1), portions of dialyzable and non-dialyzable fractions of infusions (5.0 g) were weighted into 50-mL PP digestion tubes, treated with 4 mL of concentrated HNO_3_, covered with PP glasses, placed in a digestion block, and heated at 100 °C for 2 h. After cooling, clear solutions were diluted with water to 25.0 g. In case of direct analysis (P2), portions of dialyzable and non-dialyzable fractions of tea infusions (5.0 g) were analyzed as obtained, i.e., without any dilution and/or acidification. Finally, for acidification with HNO_3_ (P3), portions of dialyzable and non-dialyzable fractions of infusions (5.0 g) were placed into 10-mL PP tubes and acidified with concentrated HNO_3_ to a concentration of 0.25 mol L^−1^. For each procedure, respective procedural blanks were prepared and included in the final results.

All samples were prepared and analyzed in triplicate (*n* = 3). Prior to FAAS measurements (determination of Ca and Mg), sample solutions were × 25 times diluted and analyzed against simple standard solutions. In case of the remaining metals, ICP OES measurements were done using undiluted sample solutions versus matrix-matching standard solutions. Mentioned matrix-matching standards were prepared on the basis of respective blank solutions to avoid differences between matrices of standards and samples.

### Statistical Analysis of Results

The one-tailed Snedecor-Fisher *F*-test with a critical parameter (*F*_critical_) at the 95% significance level (*α* = 0.05) of 19.00 was used to examine statistically significant differences between standard deviations (SDs) of mean concentrations of metals determined using the reference sample preparation procedure (P1) and two other alternative sample preparation procedures (P2, P3), indicating differences in precision of results achieved with them [26]. When calculated values of the *F*-test (*F*_calculated_) were lower than *F*_critical_, SDs of results did not statistically differ, and hence, the two-sample Student *t* test was used to compare respective mean concentrations of studied metals with a critical value (*t*_critical_) of 2.776 (*α* = 0.05) [26].

Two-side one-way analysis of variance (ANOVA) for independent groups was applied to assess differences between within-group variance and between-group variance in collected data. Because of heteroscedasticity of variance of concentrations of studied metals in infusions of analyzed BTs and GTs, the Welch test was used to determine all *F*-values. Test significance (*p* value) lower than 0.05 meant that differences between compared mean concentrations were statistically significant.

Supervised linear discriminant analysis (LDA) was used to find possible classification of analyzed BTs and GTs. LDA was carried out using two different conditions, i.e., (I) all variables were used and within-class covariance matrices were assumed to be different, and (II) backward selection algorithm was used to select statistically significant variables and within-class covariance matrices were assumed to be equal.

## Results and Discussion

### Comparison of Different Sample Preparation Procedures

The effect of sample preparation executed after enzymatic digestion of tea infusions before multi-element analysis of resultant dialyzates by FAAS and ICP OES was investigated to select the proper procedure allowing getting reliable results of the bioaccessibility in vitro assay, i.e., precise and accurate concentrations of metals in the dialyzable fraction of infusions of BTs and GTs.

Precision and accuracy of results obtained using direct analysis of dialyzates (P2) or their prior acidification with HNO_3_ (P3) were assessed by comparing respective SDs and mean concentrations of studied metals with those achieved using wet digestion (P1). The latter sample preparation procedure was selected as the reference one because it is a well-established method to provide total decomposition of sample matrices and complete release of analytes into solutions. Its adequateness was verified by comparison of sums of mean concentrations of metals determined in wet digested dialyzable and non-dialyzable fractions separated from tea infusions with total concentrations of studied metals determined in these tea infusions.

### Figures of Merit

Total concentrations of metals in infusions of BTB1 and GTB1 and sums of their concentrations in dialyzable and non-dialyzable fractions assessed using the procedure P1 in addition to calculated values of *F*- and *t* tests are given in Table 1. No statistically significant differences between SDs for sums of concentrations of metals determined in wet digested dialyzates and non-dialyzates of tea infusions and total concentrations of these metals in analyzed tea infusions (a mass balance study) were observed. Accordingly, *F*_calculated_ values were lower than the *F*_critical_ value. In addition, it was established that there were no statistically significant differences between sums of concentrations of metals in both mentioned fractions distinguished in infusions of BTB1 and GTB1 and total concentrations of metals in these infusions. In this case, calculated values of the *t* test (*t*_calculated_) for all studied metals were also lower than the *t*_critical_ value of this test, i.e., within the range of 0.314–2.582 and 0.408–2.317 for BTB1 and GTB1, respectively. This clearly indicated that wet digestion (P1) could be used as the reference sample preparation procedure.

**Table 1:** Concentrations of metals determined in infusions of bagged black tea 1 (BTB1) and bagged green tea 1 (GTB1) and their concentrations determined in dialyzable and non-dialyzable fractions after application of wet digestion (the reference sample preparation procedure P1) using FAAS (Ca, Mg) and ICP OES (Al, Ba, Cu, Fe, Mn, Ni, Sr, Zn)

	C_t_^a^	A: dialyzate^b^	B: non-dialyzate^c^	Sum (A and B)^d^	*F*_calculated_^e^	t_calculated_^f^
BTB1						
Al/10^3^	3.93 ± 0.03	0.050 ± 0.001	4.05 ± 0.08	4.10 ± 0.11	13.44	2.582
Ba	17.8 ± 0.2	4.92 ± 0.11	12.7 ± 0.2	17.6 ± 0.3	2.25	0.961
Ca/10^3^	3.08 ± 0.09	1.38 ± 0.03	1.75 ± 0.04	3.13 ± 0.07	1.65	0.760
Cu	55.1 ± 1.1	15.5 ± 0.2	39.9 ± 0.5	55.4 ± 0.8	1.89	0.382
Fe	61.9 ± 0.7	8.32 ± 0.33	53.9 ± 0.8	62.2 ± 1.5	4.59	0.314
Mg/10^3^	7.06 ± 0.07	3.01 ± 0.10	4.32 ± 0.14	7.33 ± 0.22	9.88	2.026
Mn/10^3^	1.14 ± 0.03	0.378 ± 0.02	0.753 ± 0.002	1.13 ± 0.01	9.00	0.548
Ni	21.9 ± 0.7	4.61 ± 0.15	17.8 ± 0.3	22.4 ± 0.5	1.96	1.007
Sr	6.65 ± 0.19	1.80 ± 0.08	4.93 ± 0.13	6.73 ± 0.24	1.60	0.453
Zn	97.5 ± 0.8	40.1 ± 0.8	59.9 ± 1.0	100 ± 2	6.25	2.010
GTB1						
Al/10^3^	5.10 ± 0.06	0.321 ± 0.010	4.76 ± 0.05	5.08 ± 0.06	1.00	0.408
Ba	26.7 ± 0.4	9.30 ± 0.28	18.3 ± 0.4	27.6 ± 0.6	2.25	2.162
Ca/10^3^	0.903 ± 0.012	0.397 ± 0.012	0.513 ± 0.033	0.910 ± 0.024	4.00	0.452
Cu	54.6 ± 0.4	14.9 ± 0.3	40.9 ± 0.6	55.6 ± 0.7	3.06	2.148
Fe	58.7 ± 2.6	7.47 ± 0.28	51.2 ± 2.3	58.7 ± 2.5	1.08	0.000
Mg/10^3^	4.79 ± 0.09	2.19 ± 0.03	2.78 ± 0.07	4.97 ± 0.10	1.24	2.317
Mn/10^3^	2.65 ± 0.04	0.586 ± 0.015	2.05 ± 0.02	2.64 ± 0.01	16.00	0.420
Ni	35.0 ± 1.0	5.67 ± 0.12	29.8 ± 1.3	35.5 ± 1.1	1.21	0.583
Sr	4.93 ± 0.07	1.28 ± 0.03	3.63 ± 0.03	4.91 ± 0.03	5.44	0.455
Zn	75.6 ± 0.9	25.2 ± 0.4	52.9 ± 1.6	78.1 ± 1.8	4.00	2.152

Mean values (*n* = 3) ± standard deviations. In addition, calculated values of *F*- (*F*_calculated_) and *t* (*t*_calculated_) tests are given for comparison of standard deviations and means assessed for sums of concentrations of metals determined in both fractions and total concentrations of metals determined in infusions

^a^Total concentrations of metals in tea infusions (in μg L^−1^)

^b^Concentrations of metals in the dialyzable fraction (in μg L^−1^)

^c^Concentrations of metals in the non-dialyzable fraction (in μg L^−1^)

^d^Sums of concentrations of metals in dialyzable and non-dialyzable fractions (in μg L^−1^)

^e^Calculated values of the *F*-test, *F*_critical_ = 19.00 (*α* = 0.05)

^f^Calculated values of the *t* test, *t*_critical_ = 2.776 (*α* = 0.05)

*F*_calculated_ values for comparison of SDs of results obtained for alternative sample preparation procedures (P2, P3) and those for the reference procedure (P1) are presented in Table 2. As can be seen, in all cases, they were lower than the *F*_critical_ value. It showed that differences between SDs of mean concentrations of metals (being the measure of precision of results) for compared alternative sample preparation procedures (P2 and P3) did not differ statistically from those obtained for the reference procedure (P1). For easier comparison, precision of results was expressed as relative standard deviation (%RSD) of mean concentrations of metals in the dialyzable fraction of infusions of BTB1 and GTB1. As given in Table 2, precision of results of multi-element analysis of the dialyzable fraction of infusions of BTB1 achieved with the aid of procedures P1 (0.49–4.2%) and P3 (0.80–3.9%) was comparable. In case of the procedure P2, it was slightly worse, i.e., RSDs were within 1.9–7.0%. RSDs assessed for multi-element analysis of infusions of GTB1 were quite comparable, i.e., 1.5–3.8% for the procedure P1, 1.1–7.8% for the procedure P2, and 1.4–5.0% for the procedure P3.

**Table 2:** Concentrations of metals in the dialyzable fraction of infusions of bagged black tea 1 (BTB1) and bagged green tea 1 (GTB1) determined by FAAS (Ca, Mg) and ICP OES (Al, Ba, Cu, Fe, Mn, Ni, Sr, Zn) after different sample preparation of collected dialyzates, i.e., wet digestion (P1), no prior treatment = direct analysis (P2) and acidification with HNO_3_ (P3)

	BTB1			GTB1		
	P1	P2	P3	P1	P2	P3
Concentrations (in μg L^−1^); mean values (*n* = 3) ± standard deviations						
Al/10^3^	0.050 ± 0.001	0.054 ± 0.003	0.048 ± 0.001	0.321 ± 0.010	0.322 ± 0.005	0.309 ± 0.004
Ba	4.92 ± 0.11	8.94 ± 0.20	5.04 ± 0.09	9.30 ± 0.28	13.4 ± 0.3	9.20 ± 0.46
Ca/10^3^	1.38 ± 0.03	1.58 ± 0.11	1.40 ± 0.06	0.397 ± 0.012	0.443 ± 0.019	0.416 ± 0.007
Cu	15.5 ± 0.2	15.1 ± 0.3	15.7 ± 0.2	14.9 ± 0.3	10.8 ± 0.4	15.5 ± 0.3
Fe	8.32 ± 0.33	6.55 ± 0.45	8.17 ± 0.32	7.47 ± 0.28	5.46 ± 0.39	7.16 ± 0.18
Mg/10^3^	3.01 ± 0.10	3.22 ± 0.09	2.88 ± 0.07	2.19 ± 0.03	2.32 ± 0.02	2.15 ± 0.05
Mn/10^3^	0.378 ± 0.02	0.338 ± 0.006	0.373 ± 0.003	0.586 ± 0.015	0.679 ± 0.015	0.584 ± 0.011
Ni	4.61 ± 0.15	4.13 ± 0.19	4.77 ± 0.04	5.67 ± 0.12	5.93 ± 0.20	5.46 ± 0.07
Sr	1.80 ± 0.08	0.919 ± 0.06	1.71 ± 0.03	1.28 ± 0.03	0.765 ± 0.034	1.22 ± 0.04
Zn	40.1 ± 0.8	26.7 ± 1.1	39.4 ± 0.6	25.2 ± 0.4	8.48 ± 0.30	25.9 ± 0.4
*F*_calculated_, *F*_critical_ = 19.00 (*α* = 0.05); in reference to results obtained for the reference procedure (P1)						
Al/10^3^	–	9.00	1.00	–	4.00	6.25
Ba	–	3.31	1.49	–	1.15	2.70
Ca/10^3^	–	13.44	4.00	–	2.51	2.94
Cu	–	2.25	1.00	–	1.78	1.00
Fe	–	1.86	1.06	–	1.94	2.42
Mg/10^3^	–	1.23	2.04	–	2.25	2.78
Mn/10^3^	–	9.00	2.25	–	1.00	1.86
Ni	–	1.60	14.06	–	2.78	2.94
Sr	–	1.78	7.11	–	1.28	1.78
Zn	–	1.89	1.78	–	0.56	1.00
*t*_calculated_, *t*_critical_ = 2.776 (*α* = 0.05); in reference to results obtained for the reference procedure (P1)						
Al	–	2.191	2.450	–	0.155	1.930
Ba	–	*30.505*	1.462	–	*17.305*	0.322
Ca	–	*3.038*	0.516	–	*3.546*	2.369
Cu	–	1.922	1.225	–	*14.203*	2.450
Fe	–	*5.494*	0.565	–	*7.251*	1.613
Mg	–	2.704	1.845	–	*6.245*	1.188
Mn	–	*10.954*	2.402	–	*7.593*	0.186
Ni	–	*3.434*	1.785	–	1.931	2.618
Sr	–	*15.259*	1.824	–	*19.672*	2.078
Zn	–	*17.064*	1.212	–	*57.920*	2.143

In addition, calculated values of *F*- (*F*_calculated_) and *t* (*t*_calculated_) tests are given for comparison of standard deviations and means of concentrations of metals obtained for alternative procedures (P2 and P3) with those obtained for the reference procedure (P1). Significant differences are italicized

Accuracy of results achieved with both alternative sample preparation procedures was verified by comparing mean concentrations of metals determined in the dialyzable fraction of infusions of BTB1 and GTB1 by direct analysis of dialyzates (P2) or after their prior acidification with HNO_3_ (P3) with those determined after their initial wet digestion (P1). Significance of differences between these results was tested using the *t* test (see Table 2). In reference to *t*_calculated_ values, it was established that results achieved using acidification of dialyzates with HNO_3_ (P3) did not statistically differ from those obtained with their wet digestion (P1). Accordingly, *t*_calculated_ values were lower than the *t*_critical_ value and varied within 0.516–2.450 and 0.186–2.618, respectively, for infusions of BTB1 and GTB1. Relative errors assessed for mean concentrations of metals achieved with the procedure P3 in reference to mean concentrations of metals obtained with the reference procedure P1 were changed from − 5.0 to + 3.5% and from − 4.7 to + 4.8% for infusions of BTB1 and GTB1, respectively. Considering direct analysis (P2) of infusions of BTB1 and GTB1, statistically significant differences between mean concentrations of studied metals achieved with this procedure and the reference procedure P1 were noted for a great number of studied metals, i.e., 7 (BTB1) and 8 (GTB2). Respective relative errors for results obtained with this procedure (P2) in reference to results obtained using wet digestion (P1) were changed from − 49 to + 82% (BTB1) and from − 66 to + 44% (GTB1). In view of this, direct analysis of dialyzates (P2) could not be used for reliable determination of all metals in the bioaccessible fraction of tea infusions, although it was previously applied by many researchers [4–11]. Nevertheless, in cited papers, reliability of results of such analyses was not verified.

Finally, it was considered that although sample preparation procedures P1 and P3 were adequate for assessment of the bioaccessible fraction, acidification of dialyzates of tea infusions with HNO_3_ (P3) was preferred due to its simplicity and easiness. This treatment was regarded as a very good alternative to time-consuming and laborious wet digestion (P1).

Spike-and-recovery experiments were also carried out at two concentration levels of studied metals, i.e., 25 and 50 μg L^−1^. Experiments were carried out in triplicate (*n* = 3) for all metals except for Ca and Mg, because their concentrations were much higher than others. Recoveries (see Table 3) obtained for metals in the dialyzable fraction of infusions of BTB1, prepared using sample preparation procedures P1-P3 prior to measurements by ICP OES, were within the following ranges: 97.2–106% (P1), 83.9–110% (P2), and 97.4–105% (P3) In case of infusions of GTB1, they were varied within 96.2–107% (P1), 84.3–109% (P2), and 98.0–103% (P3). Apparently, wet digestion of dialyzates (P1) and their acidification with HNO_3_ (P3) produced quantitative recoveries of all studied metals and showed the best accuracy. Additionally, slopes of calibration curves for these procedures, as achieved by standard additions and external standards, were comparable, indicating absence of any serious matrix effects. Recoveries obtained in case of direct analysis of dialyzates (P2) were in the range of 84–110% and pointed that this sample preparation procedure was inappropriate.

**Table 3:** Recoveries (in %) of metals from dialyzates of infusions of bagged black tea 1 (BTB1) and bagged green tea 1 (GTB1) prepared prior to analysis by ICP OES (Al, Ba, Cu, Fe, Mn, Ni, Sr, Zn) using different sample preparation procedures, i.e., wet digestion (P1), no prior treatment = direct analysis (P2) and acidification with HNO_3_ (P3)

	Addition^a^	BTB1			GTB1		
		P1	P2	P3	P1	P2	P3
Al	25	105 ± 1.8	108 ± 1.1	104 ± 0.2	103 ± 0.5	105 ± 0.4	101 ± 0.3
	50	104 ± 0.2	107 ± 1.4	103 ± 0.6	101 ± 0.8	106 ± 0.5	100 ± 0.1
Ba	25	102 ± 1.2	93.5 ± 1.3	99.4 ± 1.8	102 ± 0.6	95.6 ± 0.1	99.0 ± 0.5
	50	100 ± 0.2	95.9 ± 0.6	102 ± 2.1	99.8 ± 0.6	97.3 ± 0.6	98.4 ± 1.0
Cu	25	99.1 ± 0.1	103 ± 1.7	101 ± 2.9	98.8 ± 1.5	102 ± 1.6	100 ± 1.4
	50	98.6 ± 0.9	102 ± 1.5	102 ± 1.4	98.9 ± 0.3	102 ± 0.1	99.1 ± 0.2
Fe	25	97.7 ± 1.3	86.0 ± 0.3	99.9 ± 1.2	98.5 ± 0.3	88.1 ± 1.8	101 ± 1.2
	50	97.5 ± 0.6	93.3 ± 0.1	99.0 ± 0.2	97.6 ± 0.2	94.2 ± 0.8	98.2 ± 0.5
Mn	25	99.9 ± 0.6	97.8 ± 0.5	100 ± 0.4	101 ± 0.1	101 ± 0.2	101 ± 0.1
	50	99.4 ± 0.1	97.5 ± 0.6	102 ± 0.1	99.4 ± 0.7	101 ± 0.7	102 ± 0.3
Ni	25	97.2 ± 3.7	83.9 ± 0.5	97.4 ± 1.8	96.2 ± 0.3	84.3 ± 1.6	98.0 ± 0.5
	50	102 ± 0.6	97.2 ± 0.3	101 ± 0.8	99.6 ± 2.5	98.1 ± 1.7	100 ± 1.7
Sr	25	106 ± 1.4	110 ± 1.3	103 ± 1.0	107 ± 0.5	109 ± 6.0	103 ± 0.7
	50	105 ± 0.6	105 ± 0.4	105 ± 1.7	104 ± 0.5	94.2 ± 0.8	103 ± 0.6
Zn	25	101 ± 1.1	106 ± 1.3	101 ± 1.2	99.4 ± 0.6	105 ± 1.1	101 ± 0.9
	50	98.9 ± 0.3	106 ± 0.1	102 ± 1.2	97.6 ± 0.3	106 ± 0.5	98.6 ± 0.8

Mean values (*n* = 3) ± standard deviations (SDs)

^a^In μg L^−1^

LODs (3σ criterion) of studied metals evaluated for compared sample preparation procedures prior to ICP OES measurements are given in Table 4. LODs obtained for wet digestion (P1) and acidification with HNO_3_ (P3) were better than those achieved for direct analysis (P2). In general, LODs of metals assessed using procedures P1, P2, and P3 were in the range of 0.13–1.9 μg L^−1^, 0.21–2.1 μg L^−1^, and 0.075–1.1 μg L^−1^, respectively. LODs of Al, Cu, Mn, and Zn for acidification with HNO_3_ (P3) were practically the same as those obtained for wet digestion (P1). In case of Ba, Fe, Ni, and Sr, even lower LODs were achieved when the procedure P3 was used. LODs of Ca (3.3 μg L^−1^) and Mg (0.22 μg L^−1^), assessed for FAAS, were the same for all sample preparation procedures because concentrations of these metals were determined against simple standard solutions after high dilution of dialyzates.

**Table 4:** Limits of detection (LODs) of Al, Ba, Cu, Fe, Mn, Ni, Sr, Zn assessed for ICP OES combined with different sample preparation procedures of dializates of tea infusions, i.e., wet digestion (P1), no prior treatment = direct analysis (P2) and acidification with HNO_3_ (P3)

	LOD, μg L^−1^		
	P1	P2	P3
Al	0.80	2.1	0.88
Ba	0.22	0.89	0.13
Cu	0.58	0.93	0.63
Fe	1.9	1.6	1.1
Mn	0.14	0.24	0.17
Ni	0.71	1.2	0.65
Sr	0.13	0.21	0.075
Zn	0.19	0.95	0.29

Summarizing analytical performance achievable with both compared alternative sample preparation procedures, it was concluded that acidification of dialyzates of infusions of BTs and GTs with HNO_3_ (P3) ensured precise and accurate concentrations of all studied metals determined by FAAS and ICP OES. This procedure was simpler, faster, required minimal amounts of reagents, and minimized risk related to contamination of samples and loss of analytes as compared to wet digestion. In view of this, the procedure P3 was chosen as the best and used in further work, i.e., for analysis of 20 infusions of BTs and GTs that were subjected to enzymatic digestion.

## Evaluation of Bioaccessibility of Metals from Black and Green Tea Infusions

### Accuracy of the GID Procedure—a Mass Balance Study

To prove accuracy of results obtained with the aid of in vitro GID procedure for infusions of all analyzed BTs and GTs, a mass balance study was performed. For each metal, the sum of its concentrations in dialyzable and non-dialyzable fractions was compared with its total content determined in a given infusion and expressed as recovery (see Table 5). It was established that recoveries for all metals were quantitative within the following ranges: 94.7–103% and 98.7–104% (Al), 96.7–109% and 97.4–102% (Ba), 98.2–119% and 97.1–109% (Ca), 94.2–106% and 98.6–104% (Cu), 93.7–109% and 98.1–105% (Fe), 94.3–99.9% and 97.0–104% (Mg), 98.2–109% and 98.9–103% (Mn), 96.2–106% and 95.5–105% (Ni), 95.9–104% and 99.5–111% (Sr), and 100.0–109.6% and 97.8–104.0% (Zn), respectively, for infusions of BTs and GTs. Precision of measurements, expressed as %RSD was also good and varied from 0.10 to 4.5% for infusions of BTs and from 0.16 to 4.5% for infusions of GTs. Only for Ca (BTL2 and BTL4) and for Ni (GTB5 and GTL2), precision was slightly worse, i.e., from 5.7 to 6.4%.

**Table 5:** A mass balance study for in vitro gastrointestinal digestion of infusions of black (BTs) and green (GTs) teas and determination of metals after acidification of dialyzates and non-dialyzates with HNO_3_

BTs										
	BTB1	BTB2	BTB3	BTB4	BTB5	BTL1	BTL2	BTL3	BTL4	BTL5
Al/10^3^										
C_t_^a^	3.93 ± 0.03	3.30 ± 0.04	3.94 ± 0.04	2.22 ± 0.03	1.67 ± 0.01	1.75 ± 0.01	1.32 ± 0.02	3.03 ± 0.02	1.46 ± 0.02	1.97 ± 0.03
A: dialyzate^b^	0.048 ± 0.001	0.048 ± 0.001	0.042 ± 0.001	0.026 ± 0.001	0.017 ± 0.001	0.021 ± 0.001	0.018 ± 0.001	0.024 ± 0.001	0.020 ± 0.001	0.023 ± 0.001
B: non-dialyzate^c^	3.99 ± 0.08	3.28 ± 0.03	3.88 ± 0.02	2.19 ± 0.01	1.63 ± 0.01	1.67 ± 0.01	1.26 ± 0.02	2.85 ± 0.02	1.42 ± 0.01	1.88 ± 0.02
Sum (A and B)^d^	4.04 ± 0.09	3.33 ± 0.03	3.92 ± 0.02	2.22 ± 0.01	1.65 ± 0.01	1.69 ± 0.01	1.28 ± 0.02	2.87 ± 0.02	1.44 ± 0.01	1.90 ± 0.02
Agreement^e^	103 ± 2	101 ± 1	99.5 ± 0.5	100 ± 1	98.8 ± 0.7	96.6 ± 0.6	97.0 ± 1.2	94.7 ± 0.5	98.6 ± 0.8	96.6 ± 0.8
Ba										
C_t_^a^	17.8 ± 0.2	49.0 ± 0.2	27.1 ± 0.3	11.0 ± 0.2	7.28 ± 0.09	3.92 ± 0.13	5.45 ± 0.10	6.25 ± 0.05	3.38 ± 0.03	3.80 ± 0.16
A: dialyzate^b^	5.04 ± 0.09	13.5 ± 0.4	6.53 ± 0.08	3.08 ± 0.06	1.88 ± 0.03	1.28 ± 0.03	1.84 ± 0.06	2.11 ± 0.01	1.27 ± 0.01	1.04 ± 0.04
B: non-dialyzate^c^	12.9 ± 0.2	39.7 ± 0.3	21.2 ± 0.1	7.56 ± 0.02	5.31 ± 0.03	2.64 ± 0.02	3.54 ± 0.04	4.49 ± 0.08	2.02 ± 0.02	2.72 ± 0.02
Sum (A and B)^d^	17.9 ± 0.2	53.2 ± 0.6	27.7 ± 0.1	10.6 ± 0.1	7.19 ± 0.03	3.92 ± 0.02	5.38 ± 0.07	6.60 ± 0.08	3.29 ± 0.02	3.76 ± 0.05
Agreement^e^	101 ± 1	109 ± 1	102 ± 1	96.7 ± 0.3	98.8 ± 0.4	100 ± 1	98.7 ± 1.3	106 ± 1	97.3 ± 0.7	99.0 ± 1.4
Ca/10^3^										
C_t_^a^	3.08 ± 0.09	2.20 ± 0.04	2.74 ± 0.04	1.62 ± 0.02	1.67 ± 0.01	1.28 ± 0.01	1.60 ± 0.03	2.98 ± 0.07	1.75 ± 0.03	1.66 ± 0.03
A: dialyzate^b^	1.40 ± 0.05	0.915 ± 0.027	1.21 ± 0.05	0.581 ± 0.012	0.711 ± 0.026	0.536 ± 0.026	0.645 ± 0.044	1.22 ± 0.06	0.805 ± 0.055	0.461 ± 0.009
B: non-dialyzate^c^	1.82 ± 0.06	1.45 ± 0.04	2.00 ± 0.02	1.26 ± 0.06	1.27 ± 0.02	0.888 ± 0.013	1.05 ± 0.02	2.20 ± 0.02	1.24 ± 0.06	1.17 ± 0.03
Sum (A and B)^d^	3.22 ± 0.09	2.36 ± 0.05	3.21 ± 0.05	1.84 ± 0.08	1.98 ± 0.04	1.42 ± 0.05	1.70 ± 0.10	3.42 ± 0.02	2.04 ± 0.13	1.63 ± 0.03
Agreement^e^	105 ± 3	107 ± 2	117 ± 2	114 ± 5	119 ± 3	111 ± 4	106 ± 6	115 ± 1	117 ± 7	98.2 ± 1.8
Cu										
C_t_^a^	55.1 ± 1.1	41.3 ± 1.5	55.5 ± 0.5	47.7 ± 0.6	34.6 ± 1.4	36.8 ± 1.2	41.2 ± 1.1	69.6 ± 0.8	28.4 ± 0.8	29.1 ± 0.3
A: dialyzate^b^	15.7 ± 0.2	11.2 ± 0.5	15.0 ± 0.6	13.7 ± 0.5	12.6 ± 0.3	8.65 ± 0.10	7.99 ± 0.13	13.8 ± 0.5	5.57 ± 0.12	6.17 ± 0.24
B: non-dialyzate^c^	40.6 ± 0.7	29.1 ± 1.1	40.4 ± 0.9	32.1 ± 0.4	23.9 ± 0.3	26.4 ± 0.5	32.4 ± 0.7	51.8 ± 0.4	21.9 ± 0.9	23.2 ± 0.2
Sum (A and B)^d^	56.3 ± 0.8	40.3 ± 0.7	55.4 ± 0.7	45.8 ± 1.2	36.5 ± 0.4	35.0 ± 0.5	40.4 ± 0.8	65.6 ± 0.6	27.5 ± 0.8	29.4 ± 0.3
Agreement^e^	102 ± 1	97.6 ± 1.8	99.8 ± 1.2	96.0 ± 2.6	106 ± 1.2	95.1 ± 1.3	98.0 ± 2.0	94.2 ± 0.9	96.8 ± 2.9	101 ± 1
Fe										
C_t_^a^	61.9 ± 0.7	29.7 ± 1.4	77.8 ± 4.4	39.4 ± 2.2	37.9 ± 1.4	46.6 ± 0.7	33.3 ± 2.2	39.6 ± 1.6	43.8 ± 0.7	43.0 ± 1.5
A: dialyzate^b^	8.17 ± 0.32	3.83 ± 0.06	3.40 ± 0.06	4.45 ± 0.12	1.98 ± 0.04	4.37 ± 0.12	3.36 ± 0.03	3.08 ± 0.14	4.65 ± 0.03	3.45 ± 0.10
B: non-dialyzate^c^	55.5 ± 1.0	27.6 ± 0.2	77.0 ± 2.3	34.6 ± 0.7	35.2 ± 1.0	41.3 ± 0.9	30.7 ± 0.5	35.5 ± 0.6	45.8 ± 1.2	43.6 ± 1.0
Sum (A and B)^d^	63.7 ± 1.3	31.4 ± 0.2	80.4 ± 2.3	39.0 ± 0.8	37.2 ± 1.0	45.7 ± 1.0	34.1 ± 0.5	38.6 ± 0.8	50.4 ± 1.2	47.0 ± 0.8
Agreement^e^	103 ± 2	106 ± 1	103 ± 3	99.0 ± 2.1	98.2 ± 2.6	98.1 ± 2.1	102 ± 2	97.5 ± 2.1	93.7 ± 2.2	109 ± 2
Mg/10^3^										
C_t_^a^	7.06 ± 0.07	6.66 ± 0.13	9.14 ± 0.10	4.09 ± 0.02	5.33 ± 0.12	4.30 ± 0.04	6.59 ± 0.17	7.04 ± 0.16	7.44 ± 0.15	5.48 ± 0.07
A: dialyzate^b^	2.88 ± 0.07	2.42 ± 0.08	3.57 ± 0.06	1.68 ± 0.02	2.18 ± 0.03	1.80 ± 0.07	2.56 ± 0.03	2.63 ± 0.06	3.11 ± 0.07	2.25 ± 0.01
B: non-dialyzate^c^	4.04 ± 0.12	3.86 ± 0.03	5.34 ± 0.12	2.29 ± 0.05	3.14 ± 0.06	2.35 ± 0.01	3.84 ± 0.08	4.40 ± 0.20	4.30 ± 0.08	3.16 ± 0.08
Sum (A and B)^d^	6.92 ± 0.18	6.28 ± 0.08	8.91 ± 0.19	3.97 ± 0.07	5.32 ± 0.07	4.15 ± 0.06	6.40 ± 0.11	7.03 ± 0.25	7.41 ± 0.15	5.41 ± 0.08
Agreement^e^	98.0 ± 2.5	94.3 ± 1.1	97.5 ± 2.0	97.1 ± 1.7	99.8 ± 1.3	96.5 ± 1.4	97.1 ± 1.6	99.9 ± 3.6	99.6 ± 2.0	98.7 ± 1.4
Mn/10^3^										
C_t_^a^	1.14 ± 0.03	1.23 ± 0.01	2.04 ± 0.01	0.918 ± 0.007	1.24 ± 0.01	0.611 ± 0.006	1.00 ± 0.01	0.339 ± 0.005	1.22 ± 0.01	0.748 ± 0.002
A: dialyzate^b^	0.373 ± 0.003	0.391 ± 0.001	0.640 ± 0.005	0.319 ± 0.002	0.391 ± 0.002	0.211 ± 0.001	0.338 ± 0.002	0.091 ± 0.002	0.458 ± 0.002	0.257 ± 0.002
B: non-dialyzate^c^	0.750 ± 0.006	0.932 ± 0.008	1.59 ± 0.01	0.670 ± 0.002	0.893 ± 0.006	0.407 ± 0.005	0.708 ± 0.004	0.250 ± 0.002	0.854 ± 0.004	0.500 ± 0.006
Sum (A and B)^d^	1.12 ± 0.01	1.32 ± 0.01	2.23 ± 0.02	0.989 ± 0.004	1.28 ± 0.01	0.618 ± 0.004	1.05 ± 0.01	0.341 ± 0.003	1.31 ± 0.01	0.757 ± 0.004
Agreement^e^	98.2 ± 0.8	108 ± 1	109 ± 1	108 ± 1	104 ± 1	101 ± 1	105 ± 1	101 ± 1	108 ± 1	101 ± 1
Ni										
C_t_^a^	21.9 ± 0.7	27.2 ± 1.3	37.6 ± 0.3	29.9 ± 0.4	27.2 ± 0.2	23.4 ± 0.2	30.5 ± 0.9	27.4 ± 0.6	24.6 ± 1.0	21.1 ± 0.7
A: dialyzate^b^	4.77 ± 0.04	6.88 ± 0.28	7.60 ± 0.21	5.62 ± 0.10	5.06 ± 0.06	10.6 ± 0.3	11.5 ± 0.2	7.62 ± 0.12	8.76 ± 0.15	5.32 ± 0.11
B: non-dialyzate^c^	17.6 ± 0.4	21.2 ± 0.7	28.8 ± 0.5	24.2 ± 0.2	21.6 ± 0.9	13.9 ± 0.3	20.9 ± 1.0	18.8 ± 0.6	14.9 ± 0.6	15.0 ± 0.1
Sum (A and B)^d^	22.4 ± 0.4	28.1 ± 0.9	36.4 ± 0.7	29.8 ± 0.2	26.7 ± 1.0	24.5 ± 0.8	32.4 ± 1.1	26.4 ± 0.5	23.7 ± 0.6	20.3 ± 0.1
Agreement^e^	102 ± 2	103 ± 3	96.8 ± 1.9	99.7 ± 0.5	98.0 ± 3.5	105 ± 4	106 ± 4	96.4 ± 1.8	96.2 ± 2.5	96.3 ± 0.5
Sr										
C_t_^a^	6.65 ± 0.19	22.3 ± 0.1	10.7 ± 0.1	5.21 ± 0.04	4.68 ± 0.07	1.71 ± 0.03	1.92 ± 0.06	4.38 ± 0.06	3.13 ± 0.08	1.93 ± 0.04
A: dialyzate^b^	1.71 ± 0.03	6.29 ± 0.24	2.38 ± 0.10	1.27 ± 0.02	1.46 ± 0.04	0.345 ± 0.013	0.444 ± 0.012	1.41 ± 0.03	0.426 ± 0.008	0.415 ± 0.004
B: non-dialyzate^c^	4.96 ± 0.20	15.1 ± 0.2	8.71 ± 0.32	3.93 ± 0.02	3.42 ± 0.14	1.30 ± 0.05	1.50 ± 0.05	2.82 ± 0.13	2.64 ± 0.03	1.52 ± 0.03
Sum (A and B)^d^	6.67 ± 0.24	21.4 ± 0.4	11.1 ± 0.4	5.20 ± 0.01	4.88 ± 0.14	1.64 ± 0.06	1.94 ± 0.06	4.23 ± 0.10	3.07 ± 0.03	1.94 ± 0.03
Agreement^e^	100 ± 4	95.9 ± 1.7	104 ± 3	99.8 ± 0.1	104 ± 2.8	96.2 ± 3.5	101 ± 3	96.6 ± 2.3	98.0 ± 0.8	100 ± 2
Zn										
C_t_^a^	97.5 ± 0.8	65.4 ± 0.7	140 ± 1	48.4 ± 0.2	53.2 ± 1.4	50.1 ± 1.9	71.4 ± 2.4	73.8 ± 2.2	80.4 ± 0.6	59.0 ± 0.5
A: dialyzate^b^	39.4 ± 0.6	27.6 ± 0.3	55.9 ± 1.2	20.0 ± 0.5	22.6 ± 0.4	17.4 ± 0.2	21.9 ± 1.2	26.0 ± 1.0	30.5 ± 0.9	21.6 ± 0.3
B: non-dialyzate^c^	61.9 ± 2.0	44.1 ± 2.0	98.0 ± 1.9	30.8 ± 0.1	33.7 ± 0.6	32.7 ± 1.3	51.0 ± 1.7	48.8 ± 1.8	52.7 ± 1.7	39.6 ± 1.8
Sum (A and B)^d^	101 ± 3	71.7 ± 2.2	154 ± 2	50.8 ± 0.6	56.3 ± 0.8	50.1 ± 1.3	72.9 ± 2.5	74.8 ± 2.7	83.2 ± 2.5	61.2 ± 2.1
Agreement^e^	104 ± 3	110 ± 3	110 ± 1	105 ± 1	106 ± 2	100 ± 2	102 ± 3	101 ± 4	104 ± 3	104 ± 4
GTs										
	GTB1	GTB2	GTB3	GTB4	GTB5	GTL1	GTL2	GTL3	GTL4	GTL5
Al/10^3^										
C_t_^a^	5.10 ± 0.06	10.1 ± 0.1	2.91 ± 0.02	3.06 ± 0.03	4.38 ± 0.02	2.55 ± 0.02	1.57 ± 0.01	1.63 ± 0.01	1.05 ± 0.01	1.47 ± 0.01
A: dialyzate^b^	0.309 ± 0.004	0.440 ± 0.004	0.145 ± 0.003	0.137 ± 0.001	0.229 ± 0.002	0.155 ± 0.002	0.068 ± 0.001	0.055 ± 0.001	0.053 ± 0.001	0.038 ± 0.001
B: non-dialyzate^c^	4.90 ± 0.07	9.76 ± 0.15	2.85 ± 0.03	3.00 ± 0.03	4.15 ± 0.02	2.38 ± 0.03	1.48 ± 0.01	1.56 ± 0.01	1.04 ± 0.01	1.40 ± 0.01
Sum (A and B)^d^	5.21 ± 0.08	10.2 ± 0.1	3.00 ± 0.02	3.14 ± 0.03	4.38 ± 0.02	2.54 ± 0.03	1.55 ± 0.01	1.62 ± 0.01	1.09 ± 0.01	1.44 ± 0.01
Agreement^e^	102 ± 2	101 ± 1	103 ± 1	103 ± 1	100 ± 1	99.6 ± 1.3	98.7 ± 0.8	99.4 ± 0.4	104 ± 1	98.0 ± 0.9
Ba										
C_t_^a^	26.7 ± 0.4	41.8 ± 0.5	25.6 ± 0.1	18.5 ± 0.3	19.3 ± 0.5	8.57 ± 0.07	9.37 ± 0.17	8.84 ± 0.17	7.63 ± 0.21	6.64 ± 0.05
A: dialyzate^b^	9.20 ± 0.46	13.2 ± 0.1	9.82 ± 0.19	7.32 ± 0.10	6.10 ± 0.06	4.02 ± 0.01	4.75 ± 0.08	4.04 ± 0.02	3.76 ± 0.08	3.30 ± 0.05
B: non-dialyzate^c^	17.9 ± 0.2	29.5 ± 0.8	15.4 ± 0.1	11.2 ± 0.2	12.7 ± 0.1	4.49 ± 0.07	4.54 ± 0.07	4.59 ± 0.04	4.04 ± 0.06	3.28 ± 0.04
Sum (A and B)^d^	27.1 ± 0.3	42.7 ± 0.7	25.2 ± 0.1	18.5 ± 0.2	18.8 ± 0.1	8.51 ± 0.07	9.29 ± 0.15	8.63 ± 0.06	7.80 ± 0.13	6.58 ± 0.06
Agreement^e^	102 ± 1	102 ± 2	98.5 ± 0.6	100 ± 1	97.4 ± 0.5	99.3 ± 0.8	99.2 ± 1.6	97.6 ± 0.6	102 ± 2	99.1 ± 0.9
Ca/10^3^										
C_t_^a^	0.903 ± 0.012	1.48 ± 0.03	1.72 ± 0.04	2.51 ± 0.02	1.97 ± 0.01	1.87 ± 0.02	24.0 ± 0.4	2.14 ± 0.03	3.61 ± 0.03	3.53 ± 0.04
A: dialyzate^b^	0.416 ± 0.007	0.800 ± 0.038	0.950 ± 0.018	1.12 ± 0.04	0.758 ± 0.024	0.782 ± 0.012	10.7 ± 0.3	0.924 ± 0.060	1.77 ± 0.10	1.62 ± 0.02
B: non-dialyzate^c^	0.531 ± 0.011	0.761 ± 0.036	0.917 ± 0.040	1.58 ± 0.04	1.19 ± 0.03	1.25 ± 0.07	12.6 ± 0.3	1.40 ± 0.04	2.15 ± 0.07	1.94 ± 0.13
Sum (A and B)^d^	0.947 ± 0.025	1.56 ± 0.04	1.87 ± 0.08	2.70 ± 0.08	1.95 ± 0.06	2.03 ± 0.02	23.3 ± 0.8	2.32 ± 0.10	3.92 ± 0.16	3.56 ± 0.02
Agreement^e^	105 ± 3	105 ± 2	109 ± 5	108 ± 3	99.0 ± 2.9	109 ± 1	97.1 ± 3.3	108 ± 4	109 ± 4	101 ± 1
Cu										
C_t_^a^	54.6 ± 0.4	56.4 ± 1.4	82.7 ± 1.0	73.4 ± 1.1	67.0 ± 2.1	48.1 ± 1.7	41.2 ± 0.2	75.1 ± 1.1	59.1 ± 0.8	52.0 ± 0.7
A: dialyzate^b^	15.5 ± 0.3	16.3 ± 0.1	16.6 ± 0.1	18.2 ± 0.3	21.3 ± 0.7	10.2 ± 0.5	9.88 ± 0.21	13.7 ± 0.5	11.8 ± 0.5	10.6 ± 0.3
B: non-dialyzate^c^	39.5 ± 0.4	39.9 ± 1.4	65.2 ± 0.6	56.2 ± 0.8	45.8 ± 0.8	37.9 ± 0.9	32.0 ± 0.5	60.8 ± 0.3	46.5 ± 0.7	43.5 ± 1.1
Sum (A and B)^d^	55.0 ± 0.6	56.2 ± 1.4	81.8 ± 0.7	74.4 ± 1.0	67.1 ± 0.9	48.1 ± 0.4	41.9 ± 0.6	74.5 ± 0.8	58.3 ± 0.5	54.1 ± 1.4
Agreement^e^	101 ± 1	99.6 ± 2.5	98.9 ± 0.9	101 ± 1	100 ± 1	100 ± 1	102 ± 2	99.2 ± 1.1	98.6 ± 0.9	104 ± 3
Fe										
C_t_^a^	58.7 ± 2.6	52.2 ± 2.9	51.2 ± 2.9	52.8 ± 0.7	31.2 ± 0.7	62.5 ± 1.4	59.4 ± 4.3	42.5 ± 0.7	32.3 ± 2.2	64.0 ± 2.2
A: dialyzate^b^	7.16 ± 0.18	6.44 ± 0.06	5.46 ± 0.10	5.45 ± 0.04	4.52 ± 0.31	7.33 ± 0.37	6.66 ± 0.02	5.36 ± 0.08	5.05 ± 0.14	5.14 ± 0.05
B: non-dialyzate^c^	53.7 ± 1.6	47.6 ± 0.9	48.0 ± 1.2	46.4 ± 0.7	27.0 ± 0.3	54.2 ± 0.6	52.4 ± 1.0	38.0 ± 0.9	28.9 ± 0.4	57.7 ± 0.8
Sum (A and B)^d^	60.9 ± 1.6	54.0 ± 0.9	53.5 ± 1.2	51.8 ± 0.8	31.5 ± 0.4	61.5 ± 1.0	59.1 ± 1.0	43.4 ± 1.0	34.0 ± 0.4	62.8 ± 0.7
Agreement^e^	104 ± 3	103 ± 2	104 ± 2	98.1 ± 1.5	101 ± 1.4	98.4 ± 1.7	99.5 ± 1.7	102 ± 2	105 ± 1	98.1 ± 1.1
Mg/10^3^										
C_t_^a^	4.79 ± 0.09	7.44 ± 0.03	4.48 ± 0.07	5.33 ± 0.02	5.56 ± 0.03	5.47 ± 0.04	7.50 ± 0.03	4.16 ± 0.02	4.87 ± 0.04	5.66 ± 0.06
A: dialyzate^b^	2.15 ± 0.05	3.02 ± 0.11	2.10 ± 0.08	2.27 ± 0.02	2.47 ± 0.05	2.31 ± 0.10	3.41 ± 0.09	1.74 ± 0.02	1.98 ± 0.09	2.58 ± 0.06
B: non-dialyzate^c^	2.77 ± 0.06	4.30 ± 0.09	2.58 ± 0.04	2.94 ± 0.06	3.05 ± 0.08	3.39 ± 0.07	4.15 ± 0.02	2.32 ± 0.05	3.03 ± 0.06	2.91 ± 0.05
Sum (A and B)^d^	4.92 ± 0.05	7.32 ± 0.16	4.68 ± 0.13	5.21 ± 0.05	5.52 ± 0.14	5.70 ± 0.16	7.56 ± 0.10	4.06 ± 0.05	5.01 ± 0.22	5.49 ± 0.08
Agreement^e^	103 ± 1	98.4 ± 2.2	104 ± 3	97.8 ± 0.9	99.3 ± 2.5	104 ± 3	101 ± 2	97.6 ± 1.2	103 ± 4.5	97.0 ± 1.6
Mn/10^3^										
C_t_^a^	2.65 ± 0.04	4.52 ± 0.04	1.93 ± 0.03	2.22 ± 0.02	2.00 ± 0.03	0.353 ± 0.013	2.47 ± 0.02	1.25 ± 0.02	1.67 ± 0.03	1.70 ± 0.01
A: dialyzate^b^	0.584 ± 0.011	0.925 ± 0.005	0.639 ± 0.004	0.676 ± 0.004	0.584 ± 0.003	0.133 ± 0.001	0.841 ± 0.005	0.332 ± 0.001	0.604 ± 0.002	0.509 ± 0.002
B: non-dialyzate^c^	2.09 ± 0.01	3.72 ± 0.04	1.31 ± 0.01	1.55 ± 0.01	1.41 ± 0.01	0.216 ± 0.002	1.62 ± 0.01	0.910 ± 0.005	1.12 ± 0.01	1.20 ± 0.01
Sum (A and B)^d^	2.67 ± 0.02	4.64 ± 0.04	1.95 ± 0.02	2.23 ± 0.01	1.99 ± 0.01	0.349 ± 0.003	2.46 ± 0.01	1.24 ± 0.01	1.72 ± 0.01	1.71 ± 0.01
Agreement^e^	101 ± 1	103 ± 1	101 ± 1	100 ± 1	99.5 ± 0.7	98.9 ± 0.9	99.6 ± 0.5	99.2 ± 0.3	103 ± 1	101 ± 1
Ni										
C_t_^a^	35.0 ± 1.0	71.2 ± 1.0	38.2 ± 0.7	39.6 ± 0.7	45.5 ± 0.9	24.0 ± 0.8	23.2 ± 0.1	41.6 ± 0.6	35.9 ± 0.7	32.4 ± 1.5
A: dialyzate^b^	5.46 ± 0.07	15.2 ± 0.7	3.86 ± 0.02	5.76 ± 0.14	11.6 ± 0.5	2.79 ± 0.06	2.28 ± 0.14	6.55 ± 0.22	3.25 ± 0.16	3.48 ± 0.15
B: non-dialyzate^c^	30.4 ± 0.8	52.8 ± 1.0	32.8 ± 1.5	35.8 ± 1.2	36.0 ± 2.6	20.9 ± 1.1	20.2 ± 1.2	33.4 ± 1.1	33.0 ± 0.3	29.3 ± 0.7
Sum (A and B)^d^	35.9 ± 0.7	68.0 ± 1.1	36.7 ± 1.5	41.6 ± 1.3	47.6 ± 2.7	23.7 ± 1.0	22.5 ± 1.3	40.0 ± 1.3	36.2 ± 0.3	32.8 ± 0.7
Agreement^e^	103 ± 2	95.5 ± 1.6	96.1 ± 3.9	105 ± 3	105 ± 6	98.8 ± 4.1	97.0 ± 5.7	96.2 ± 3.2	101 ± 1	101 ± 2
Sr										
C_t_^a^	4.93 ± 0.07	12.1 ± 0.3	9.52 ± 0.17	7.01 ± 0.08	7.67 ± 0.19	9.44 ± 0.09	15.4 ± 0.1	4.47 ± 0.07	4.02 ± 0.10	4.35 ± 0.03
A: dialyzate^b^	1.22 ± 0.04	3.12 ± 0.12	2.23 ± 0.10	1.86 ± 0.12	1.55 ± 0.06	4.43 ± 0.07	7.86 ± 0.33	2.04 ± 0.06	2.07 ± 0.02	2.16 ± 0.015
B: non-dialyzate^c^	3.72 ± 0.02	9.59 ± 0.32	7.24 ± 0.24	5.22 ± 0.07	6.11 ± 0.08	5.85 ± 0.20	8.11 ± 0.05	2.59 ± 0.02	2.41 ± 0.09	2.40 ± 0.06
Sum (A and B)^d^	4.94 ± 0.05	12.7 ± 0.2	9.47 ± 0.26	7.08 ± 0.08	7.66 ± 0.12	10.3 ± 0.2	16.0 ± 0.3	4.63 ± 0.06	4.48 ± 0.07	4.56 ± 0.05
Agreement^e^	100 ± 1	105 ± 2	99.5 ± 2.7	101 ± 1	99.9 ± 1.6	109 ± 3	104 ± 2	104 ± 2	111 ± 2	105 ± 1
Zn										
C_t_^a^	75.6 ± 0.9	52.1 ± 0.2	81.5 ± 0.8	98.0 ± 1.4	81.6 ± 1.5	117 ± 1	93.2 ± 0.8	101 ± 1	102 ± 1	86.4 ± 0.5
A: dialyzate^b^	25.9 ± 0.4	14.6 ± 0.4	28.8 ± 1.5	34.2 ± 0.6	30.2 ± 1.1	36.5 ± 1.1	29.6 ± 0.6	27.7 ± 0.2	26.6 ± 0.1	28.0 ± 0.6
B: non-dialyzate^c^	48.7 ± 2.2	39.2 ± 2.2	53.3 ± 2.0	66.7 ± 1.9	52.2 ± 0.8	84.4 ± 2.2	67.3 ± 0.9	71.1 ± 0.2	76.1 ± 3.0	56.8 ± 0.5
Sum (A and B)^d^	74.6 ± 2.2	53.8 ± 2.2	82.1 ± 0.7	101 ± 2.0	82.4 ± 1.7	121 ± 1.7	96.9 ± 1.2	98.8 ± 0.2	103 ± 3	84.8 ± 0.9
Agreement^e^	98.7 ± 2.9	103 ± 4	101 ± 1	103 ± 2	101 ± 2	103 ± 1.4	104 ± 1	97.8 ± 0.2	101 ± 3	98.2 ± 1.1

Mean values (*n* = 3) ± standard deviations

*BTB* bagged black tea, *BTL* leaf black tea, *GTB* bagged green tea, *GTL* leaf green tea

^a^Total concentrations of metals in tea infusions (in μg L^−1^)

^b^Concentrations of metals in the dialyzable fraction (in μg L^−1^)

^c^Concentrations of metals in the non-dialyzable fraction (in μg L^−1^)

^d^Sums of concentrations of metals in dialyzable and non-dialyzable fractions (in μg L^−1^)

^e^In %, calculated as 100 × [sum (A and B)]/*C*_*t*_

### Bioaccessibility of Metals from Black and Green Tea Infusions

**Fig. 1:**
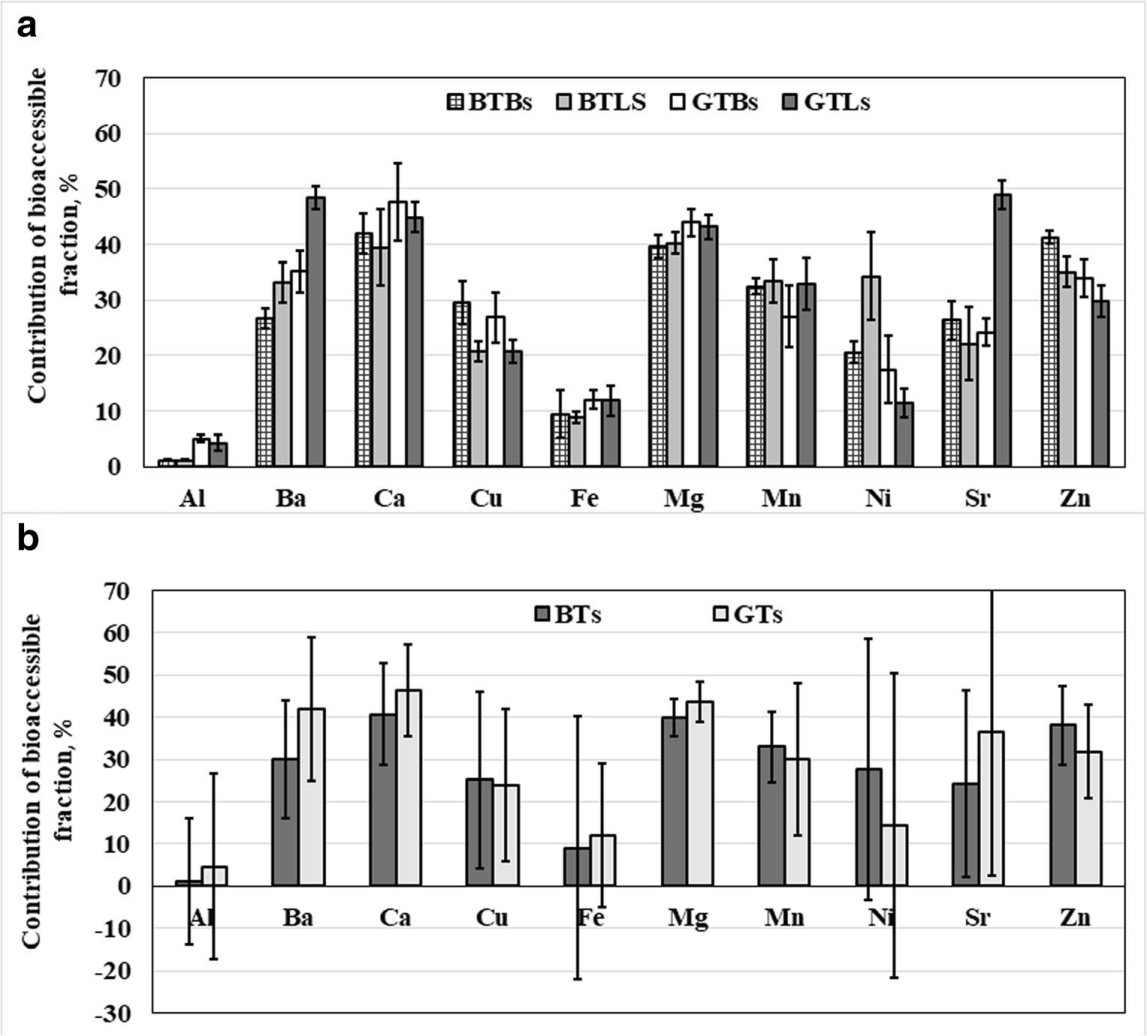
Mean contribution of the bioaccessible fraction (in %) of determined metals for infusions of different types of tea: bagged black teas (BTBs), leaf black teas (BTLs), bagged green teas (GTBs) and leaf green teas (GTLs) (a) and black teas (BTs) and green teas (GTs) (b)

Percentage contributions of the bioaccessible fraction of studied metals were calculated using the following formula: 100% × *A*/*C*_*t*_, where *A* is the concentration of a certain metal determined in the dialyzable fraction of tea infusions and *C*_*t*_—its total concentration in these infusions. Results for all analyzed teas are given in Table 6. Mean contributions of the bioaccessible fraction of Al, Ba, Ca, Cu, Fe, Mg, Mn, Ni, Sr, and Zn assessed for infusions of different types of tea (BTBs, BTLs, GTBs and GTLs) together with respective coefficients of variance (CVs) are presented in Fig. 1a. It was established that, except for Al, Mn, and Mg, bioaccessibility of studied metals from infusions of BTLs was lower by about 6% (Fe, Ca), 15% (Sr, Zn), and 30% (Cu) than bioaccessibility of these metals from infusions of BTBs. In case of Ba and Ni, contributions of the bioaccessible fraction of these metals for infusions of BTLs were higher by about 25 and 65%, respectively, than those established for infusions of BTBs. Comparing bioaccessibilities of certain metals from infusions of GTBs and GTLs, it was found that these assessed for infusions of GTLs were lower by about 6% (Ca), 10% (Zn), 15% (Al), 25% (Cu), and 35% (Ni) or higher by about 20% (Mn), 40% (Ba), and 100% (Sr) than those evaluated for infusions of GTBs.

**Table 6:** Bioaccessibility of metals from infusions of black (BTs) and green (GTs) teas

Contribution of the bioaccessible fraction, %										
Infusions of BTs										
	BTB1	BTB2	BTB3	BTB4	BTB5	BTL1	BTL2	BTL3	BTL4	BTL5
Al	1.22 ± 0.02	1.46 ± 0.03	1.07 ± 0.02	1.17 ± 0.06	1.02 ± 0.03	1.20 ± 0.04	1.36 ± 0.02	0.792 ± 0.049	1.37 ± 0.01	1.17 ± 0.06
Ba	28.3 ± 0.5	27.6 ± 0.7	24.1 ± 0.3	28.0 ± 0.5	25.8 ± 0.4	32.6 ± 0.8	33.8 ± 1.1	33.8 ± 0.2	37.6 ± 0.3	27.4 ± 1.1
Ca	45.4 ± 1.8	41.6 ± 1.9	44.2 ± 1.8	35.9 ± 0.8	42.6 ± 1.6	41.9 ± 2.5	40.3 ± 3.6	40.9 ± 2.4	46.0 ± 3.1	27.8 ± 0.5
Cu	28.5 ± 1.1	27.1 ± 2.3	27.0 ± 1.2	28.7 ± 2.0	36.4 ± 0.9	23.5 ± 0.2	19.4 ± 0.3	19.8 ± 0.7	19.6 ± 0.4	21.2 ± 0.8
Fe	13.2 ± 0.5	12.9 ± 0.1	4.37 ± 0.08	11.3 ± 0.3	5.22 ± 0.10	9.38 ± 0.55	10.1 ± 0.1	7.78 ± 0.65	8.64 ± 0.06	8.02 ± 0.23
Mg	40.8 ± 0.9	36.3 ± 1.1	39.1 ± 0.6	41.1 ± 0.4	40.9 ± 0.6	41.9 ± 1.6	38.8 ± 0.4	37.4 ± 0.9	41.8 ± 0.9	41.1 ± 0.3
Mn	32.5 ± 0.1	31.8 ± 0.1	31.4 ± 0.2	34.8 ± 0.2	31.5 ± 0.1	34.5 ± 0.1	33.8 ± 0.2	26.8 ± 0.5	37.5 ± 0.1	34.4 ± 0.2
Ni	22.7 ± 0.2	25.3 ± 1.5	20.2 ± 0.6	18.8 ± 0.3	18.6 ± 0.2	45.3 ± 2.6	37.7 ± 0.8	27.8 ± 0.5	35.6 ± 0.6	25.2 ± 0.5
Sr	25.7 ± 0.5	28.2 ± 1.1	22.2 ± 1.2	24.4 ± 0.3	31.2 ± 0.8	20.2 ± 0.7	23.1 ± 0.6	32.2 ± 0.7	13.6 ± 0.2	21.5 ± 0.2
Zn	40.4 ± 0.6	42.2 ± 0.5	39.9 ± 0.9	41.3 ± 1.1	42.5 ± 0.8	34.7 ± 0.3	30.7 ± 1.7	35.2 ± 1.3	37.9 ± 1.1	36.6 ± 0.6
Infusions of GTs										
	GTB1	GTB2	GTB3	GTB4	GTB5	GTL1	GTL2	GTL3	GTL4	GTL5
Al	6.06 ± 0.08	4.36 ± 0.04	4.98 ± 0.10	4.48 ± 0.01	5.23 ± 0.04	6.08 ± 0.07	4.30 ± 0.07	3.37 ± 0.03	5.05 ± 0.11	2.61 ± 0.04
Ba	34.5 ± 1.7	31.6 ± 0.2	38.4 ± 0.7	39.6 ± 0.6	31.6 ± 0.3	46.9 ± 0.1	50.7 ± 0.9	45.7 ± 0.3	49.3 ± 1.1	49.7 ± 0.7
Ca	46.1 ± 0.8	54.0 ± 2.6	55.2 ± 1.0	44.6 ± 1.7	38.5 ± 1.2	41.8 ± 0.6	44.6 ± 1.3	43.2 ± 2.6	49.0 ± 3.2	45.9 ± 0.6
Cu	28.4 ± 0.4	28.9 ± 0.1	20.1 ± 0.1	24.8 ± 0.5	31.8 ± 1.0	21.2 ± 1.0	24.0 ± 0.5	18.2 ± 0.7	20.0 ± 0.8	20.4 ± 0.6
Fe	12.2 ± 0.3	12.3 ± 0.1	10.7 ± 0.2	10.3 ± 0.1	14.5 ± 1.0	11.7 ± 0.6	11.2 ± 0.1	12.6 ± 0.2	15.6 ± 0.4	8.03 ± 0.08
Mg	44.9 ± 1.1	40.6 ± 1.5	46.9 ± 1.7	42.6 ± 0.4	44.4 ± 1.0	42.2 ± 1.8	45.5 ± 1.2	41.8 ± 0.5	40.7 ± 1.8	45.6 ± 1.1
Mn	22.0 ± 0.4	20.5 ± 0.1	33.1 ± 0.2	30.4 ± 0.2	29.2 ± 0.2	37.7 ± 0.3	34.0 ± 0.2	26.6 ± 0.1	36.2 ± 0.1	29.9 ± 0.1
Ni	15.6 ± 0.2	21.4 ± 1.0	10.1 ± 0.1	14.6 ± 0.3	25.5 ± 1.1	11.6 ± 0.3	9.83 ± 0.64	15.8 ± 0.5	9.05 ± 0.45	10.7 ± 0.4
Sr	24.8 ± 0.9	25.8 ± 1.0	23.4 ± 1.0	26.5 ± 1.8	20.2 ± 0.8	46.9 ± 0.7	50.9 ± 2.1	45.6 ± 1.4	51.5 ± 0.5	49.7 ± 0.1
Zn	34.3 ± 0.5	28.0 ± 0.8	35.3 ± 1.9	34.9 ± 0.7	37.0 ± 1.3	31.2 ± 1.0	31.8 ± 0.6	27.4 ± 0.3	26.1 ± 0.1	32.4 ± 0.7

Mean values (*n* = 3) ± standard deviations (SDs)

*BTB* bagged black tea, *BTL* leaf black tea, *GTB* bagged green tea, *GTL* leaf green tea

Generally, contributions of the bioaccessible fraction of metals assessed for infusions of GTs were lower than those determined for infusions of BTs for Cu (by 5%), Mn (by 9%), Zn (by 16%), and Ni (by 50%) or higher as in case of Mg (by 9%), Ca (by 15%), Fe (by 30%), Ba (by 40%), Sr (by 50%), and Al (by 300%) (see Fig. 1b). Descending orders of mean contributions of the bioaccessible fraction of studied metals in infusions of BTs and GTs were quite different, i.e., Ca > Mg > Zn > Mn > Ba>Ni > Cu > Sr > Fe > Al for BTs and Ca > Mg > Ba>Sr > Zn > Mn > Cu > Ni > Fe > Al for GTs. Differences observed in bioaccessibility of metals from both types of tea were mostly attributed to differences in manufacturing processes and composition of organic matrices of BTs and GTs. In case of BTs, due to fermentation of leaves, it contained more complex polyphenols that easier and stronger bound metals, e.g., Al, Ba, Fe, and Sr, making them insoluble during brewing of this type of tea.

Except for Ca, contributions of the bioaccessible fraction of all studied metals for infusions of BTs and GTs were lower than 50% (see Table 6). Bioaccessibility of Ca was within 27.8–46.0% (with the mean of 40.7% and CV of 12%) for infusions of BTs and 38.5–55.2% (with the mean of 46.3% and CV of 11%) for infusions of GTs. These results were quite comparable to this (47.7%) reported for infusions of BT by Powell et al. [22]. Absorption of Ca could be reduced by soluble oxalates present in infusions, which normally form insoluble salts with this metal [27]. Moreover, Brzezicka-Cirocka et al. [28] reported that the content of oxalates in BTs was higher than those in GTs, hence, it could additionally explain differences in results on bioaccessibility of Ca from infusions of BTs and GTs observed here. Mean contribution of the bioaccessible fraction of Mg was slightly lower than this for Ca and equaled to 39.9% (CV of 4.5%) for infusions of BTs and 43.5% (CV of 4.8%) for infusions of GTs. These results were seemingly lower than those for infusions of other BTs (by ~ 40% as reported by Powell et al. [22] and Erdemir [25]), herbal teas (by ~ 40% as reported by Szentmihalyli et al. [15] and ~ 20% as reported by Pereira Junior et al. [29]), and GTs (the smallest, ~ 5% difference was found as reported by Erdemir [25]). Such differences could probably be caused by compounds potentially inhibiting Mg absorption, such as oxalates and polyphenols that commonly form poorly soluble complexes with this metal in a small intense. Because tea products, particularly BTs of different origin, could contain differentiated levels of oxalates and polyphenols, bioaccessibility of Mg in infusions of the same kind of tea, particularly BTs, could vary in a quite high range. Bioaccessibility of Zn was moderate and ranged within 30.7–42.2% (with the mean of 38.1% and CV of 9.4%) for infusions of BTs and 26.1–37.0% (with the mean of 31.8% and CV of 11%) for infusions of GTs. These results were in good agreement with those reported by Powell et al. [22] for the infusion of BT, but lower than those obtained for herbal teas [15, 29]. Bioaccessibility of Zn could also be associated with chemical composition of teas because this metal can form insoluble compounds with phytates, polyphenols, and oxalic acid, which significantly reduce its solubility and absorption [4]. Contribution of the bioaccessible fraction of Mn was lower by about 14% (infusions of BTs) and 6% (infusions of GTs) as compared to this obtained for Zn and varied within 26.8–37.5% (with the mean of 32.9% and CV of 8.2%) for infusions of BTs and 20.5–37.7% (with the mean of 30.0% and CV of 18%) for infusions of GTs. Relatively low bioaccessibility of this metal could be due to the presence of phytates, ascorbic acid, and polyphenols. Additionally, because Mn is an acid-soluble metal, it could form insoluble hydroxide precipitates under conditions of GID [22]. Obtained results were slightly lower than the value of 39.8% reported by Powell et al. [22] for the infusion of BT, but in good agreement with this obtained for decoctions of cat’s claw tea [29]. Mean contribution of the bioaccessible fraction of Cu in infusions of BTs and GTs was practically the same, i.e., 25.1% (CV of 21%) and 23.8% (CV of 18%), respectively. Similarly to other metals, i.e., Fe and Zn, relatively low bioaccessibility of Cu from infusions of BTs and GTs was probably caused by composition of these teas and the presence of various endogenous ligands willingly complexing this metal, in particular phytates [30].

For metals such as Ba and Sr, significant differences in contributions of their bioaccessible fraction in infusions of BTs and GTs were observed, i.e., Ba [29.9% (CV of 14%) for infusions of BTs and 41.8% (CV of 17%) for infusions of GTs] and Sr [24.2% (CV of 22%) for infusions of BTs and 36.5% (CV of 34%) for infusions of GTs]. In our earlier study on chemical fractionation of selected metals in infusions of BTs and GTs [31], it was found that contributions of the residual fraction (RF) of Ba and Sr species in infusions of GTs were 2- and 5-fold higher, respectively, as compared to these in infusions of BTs. Such variability in results on the RF of Ba and Sr could be related to higher concentrations of organic acids in GTs than in BTs and, as a result, higher contributions of complexes of these organicals with ions of both metals in infusions of GTs rather than BTs. In case of Ni, contribution of the bioaccessible fraction was in the range of 18.6–45.3% (with the mean of 27.7% and CV of 31%) for infusions of BTs and 9.0–25.5% (with the mean of 14.4% and CV of 36%) for infusions of GTs. These results clearly indicated that infusions of BTs and GTs considerably differed due to their chemical composition. Unfortunately, it was not possible to compare results on bioaccessibility of Ba, Sr, and Ni from infusions of BTs and GTs obtained here with results of other studies because, to our best knowledge, this was the first contribution on bioaccessibility of these metals from infusions of BTs and GTs after in vitro GID. Bioaccessibility of Fe was relatively low, i.e., 4.4–13.2% (with the mean of 9.1% and CV of 31%) for infusions of BTs and 8.0–15.6% (with the mean of 11.9% and CV of 17%) for infusions of GTs. These results well corresponded to a general trend noted for Fe, but were higher than those reported by Powell et al. [22] for BT, but lower than those determined by Erdemir [25] for BTs and GTs, or Szentmihalyli et al. [15] and Pereira Junior et al. [29] for herbal teas. Such variability in results achieved for Fe could again be related to differences in composition of analyzed teas, as well as dissimilar approaches taken to simulate absorption in a small intestine, including different compositions of SGJs and SIJs. Contribution of the bioaccessible fraction of Al varied within 0.8–1.5% (with the mean of 1.2% and CV of 15%) for infusions of BTs and 2.6–6.1% (with the mean of 4.6% and CV of 22%) for infusions of GTs. Despite high concentrations of Al in infusions of analyzed teas, it was found that only a small part of this metal was available for absorption in the gastrointestinal tract. These results were in good agreement with results of studies undertaken by other authors for infusions of different BTs and GTs [22, 23, 32, 33]. Low bioaccessibility of Fe and Al from infusions of BTs and GTs was likely attributed to action of polyphenols present in their infusions, which avidly bind trivalent ions of Al and Fe and prevent their intestinal absorption [22]. Moreover, phytates and Ca at higher concentrations in teas could inhibit absorption of these metals [27]. In our previous study [31], it was established that bioaccessibility of Fe and Al from infusions of BTs and GTs (in reference to the presence of cationic fraction (CF) species) was significantly lower due to much high contributions of other species belonging to the hydrophobic fraction (HF, hydrophobic species associated with high molecular weight compounds) and the RF (neutral and/or negatively charged species associated with low and moderate weight compounds).

### Contribution of Infusions of Teas to Recommended Dietary Intakes of Metals

To estimate the nutritive value of infusions of examined BTs and GTs, mean concentrations of metals determined in the dialyzable fraction were compared with their recommended dietary intakes (RDIs), i.e., recommended dietary allowances and adequate intakes as given by National Research Council [34] for male and female in the 31–50 year life stage group. Respective RDIs (in mg day^−1^) were as follows: 1000 (Ca), 0.9 (Cu), 8–18 (Fe), 420–320 (Mg), 2.3–1.8 (Mn), and 11–8 (Zn). Considering results on bioaccessibility of metals from infusions of BTs and GTs, it appeared that consumption of four cups (1 L) of these beverages per day slightly covered RDIs of Ca, Cu, Fe, Mg, and Zn. In case of infusions of BTs, it was on average 0.09% of Ca, 1.2% of Cu, 0.05% and 0.02% of Fe (for male and female, respectively), 0.60% and 0.78% of Mg (for male and female, respectively), and 0.26% and 0.35% of Zn (for male and female, respectively). In case of infusions of GTs, it was 0.20% of Ca, 1.6% of Cu, 0.07% and 0.03% of Fe (for male and female, respectively), 0.57% and 0.75% of Mg (for male and female, respectively), and 0.26% and 0.35% of Zn (for male and female, respectively). In case of Mn, its concentration in the dialyzable fraction was established to contribute to the highest RDI realization, i.e., 15.1–19.3% and 25.4–32.4% for infusions of BTs and GTs, respectively. All these values indicated that infusions of BTs and GTs are insignificant sources of Ca, Cu, Fe, Mg, and Zn. Only for Mn, drinking of infusions of both types of tea can contribute to relatively high coverage of the RDI for this metal.

For other metals, i.e., Al, Ba, Ni, and Sr, their RDIs are not established. In case of Al, it was suggested that its high content in the human body may be related to Alzheimer disease [35]. Thus, for this metal, the Joint FAO/WHO Expert Committee on Food Additives [36] recommended the provisional tolerable weekly intake (PTWI) of 1 mg kg^−1^ body weight (b.w.). It appeared that consumption of four cups (1 L) of infusions of BTs and GTs per day by a person weighted 65 kg resulted in realization of the PTWI for Al in 0.31% and 1.8%, respectively. For Ni, the PTWI value was established as 35 μg kg^−1^ b.w. [37]. Therefore, drinking of 1 L of tea infusions could contribute to 2.3% of the PTWI of this metal in case of BTs and 1.8% for GTs. These results clearly indicated that drinking of infusions of BTs and GTs is not hazardous for human health.

### Statistical Analysis

All mean total concentrations of studied metals in infusions and in dialyzable and non-dialyzable fractions as well as percentage contributions of the dialyzable fraction distinguished from four types of tea (BTBs, BTLs, GTBs, and GTLs), along with *F*-values and *p* values are given in Table 7. To recognize differences between four groups of analyzed teas, the Fisher least significant difference (LSD) test was used. Statistically significant contrasts between certain groups of teas are given in Table 7 as well.

**Table 7:** Results of one-way ANOVA for independent groups: comparison of total concentrations of Al, Ba, Ca, Cu, Fe, Mg, Mn, Ni, Sr, and Zn in infusions of bagged black and green teas (BTBs and GTBs) and leaf black and green teas (BTLs and GTLs) in addition to concentrations of these metals in dialyzable and non-dialyzable fractions separated from infusions

	BTBs	BTLs	GTBs	GTLs	F^a^	*p* value	Post hoc differences (with *p* values)^b^
Total concentrations (mean values ± standard deviations)							
Al	3012 ± 1026	1906 ± 677	5110 ± 2936	1654 ± 550	3.61	0.062	BTLs-GTBs (0.006), GTBs-GTLs (0.004)
Ba	22.44 ± 16.65	4.56 ± 1.23	26.38 ± 9.36	8.21 ± 1.08	14.30	0.001	BTBs-BTLs (0.009), BTBs-GTLs (0.032), BTLs-GTBs (0.002), GTBs-GTLs (0.008)
Ca	2262 ± 645	1854 ± 654	1717 ± 594	7030 ± 9519	1.00	0.438	–
Cu	46.84 ± 9.01	41.02 ± 16.85	66.82 ± 11.76	55.10 ± 12.92	3.44	0.067	BTBs-GTBs (0.027), BTLs-GTBs (0.006)
Fe	49.34 ± 19.89	43.26 ± 7.66	49.22 ± 10.49	52.14 ± 14.03	0.62	0.623	–
Mg	6456 ± 1902	6170 ± 1277	5520 ± 1155	5532 ± 1246	0.44	0.727	–
Mn	1314 ± 426	784 ± 341	2664 ± 1075	1489 ± 773	4.98	0.029	BTBs-GTBs (0.009), BTLs-GTBs (0.001), GTBs-GTLs (0.020)
Ni	28.76 ± 5.73	25.40 ± 3.64	45.90 ± 14.65	31.42 ± 7.86	3.15	0.084	BTBs-GTBs (0.008), BTLs-GTBs (0.002), GTBs-GTLs (0.021)
Sr	9.91 ± 7.32	2.61 ± 1.14	8.25 ± 2.71	7.54 ± 4.93	7.16	0.013	BTBs-BTLs (0.025)
Zn	80.90 ± 38.18	66.94 ± 12.19	77.76 ± 16.59	99.92 ± 11.46	5.79	0.019	BTLs-GTLs (0.034)
Concentrations in the dialyzable fraction (mean values ± standard deviations)							
Al	36.20 ± 14.01	21.20 ± 2.39	252 ± 126	73.80 ± 46.62	7.92	0.013	BTBs-GTBs (0.000), BTLs-GTBs (0.000), GTBs-GTLs (0.001)
Ba	6.01 ± 4.55	1.51 ± 0.45	9.13 ± 2.72	3.97 ± 0.53	27.26	0.000	BTBs-BTLs (0.017), BTLs-GTBs (0.000), GTBs-GTLs (0.007)
Ca	963 ± 340	733 ± 301	809 ± 262	3159 ± 4237	0.81	0.519	–
Cu	13.64 ± 1.81	8.44 ± 3.25	17.58 ± 2.30	11.24 ± 1.56	10.59	0.003	BTBs-BTLs (0.003), BTBs-GTBs (0.016), BTLs-GTBs (0.000), GTBs-GTLs (0.000)
Fe	4.37 ± 2.31	3.78 ± 0.68	5.81 ± 1.02	5.91 ± 1.03	6.30	0.015	BTLs-GTBs (0.037), BTLs-GTLs (0.029)
Mg	2546 ± 718	2470 ± 485	2402 ± 374	2404 ± 647	0.06	0.981	–
Mn	422 ± 125	271 ± 138	682 ± 142	484 ± 269	6.41	0.014	BTBs-GTBs (0.035), BTLs-GTBs (0.002)
Ni	6.03 ± 1.16	8.76 ± 2.45	8.38 ± 4.81	3.67 ± 1.67	4.88	0.031	BTLs-GTLs (0.013), GTBs-GTLs (0.020)
Sr	2.62 ± 2.09	0.61 ± 0.45	2.00 ± 0.73	3.71 ± 2.53	6.12	0.019	BTLs-GTLs (0.011)
Zn	33.10 ± 14.76	23.48 ± 4.96	26.74 ± 7.42	29.68 ± 3.96	1.60	0.261	–
Concentrations in the non-dialyzable fraction (mean values ± standard deviations)							
Al	2994 ± 1045	1816 ± 625	4932 ± 2828	1572 ± 493	3.88	0.054	BTLs-GTBs (0.006), GTBs-GTLs (0.004)
Ba	17.33 ± 13.92	3.08 ± 0.95	17.34 ± 7.26	4.19 ± 0.55	7.56	0.011	BTBs-BTLs (0.011), BTBs-GTLs (0.018), BTLs-GTBs (0.011), GTBs-GTLs (0.018)
Ca	1560 ± 334	1310 ± 515	996 ± 405	3868 ± 4895	2.05	0.182	–
Cu	33.22 ± 7.26	31.14 ± 12.24	49.32 ± 11.15	44.14 ± 10.84	3.03	0.088	BTBs-GTBs (0.028), BTLs-GTBs (0.015)
Fe	45.98 ± 20.23	39.38 ± 6.19	44.54 ± 10.20	46.24 ± 12.26	0.57	0.648	–
Mg	3734 ± 1132	3610 ± 858	3128 ± 679	3160 ± 674	0.56	0.658	–
Mn	967 ± 364	544 ± 240	2016 ± 999	1013 ± 515	4.24	0.044	BTBs-GTBs (0.014), BTLs-GTBs (0.001), GTBs-GTLs (0.018)
Ni	22.68 ± 4.15	16.70 ± 3.00	37.56 ± 8.83	27.36 ± 6.42	9.66	0.004	BTBs-GTBs (0.001), BTLs-GTBs (0.000), BTLs-GTLs (0.013), GTBs-GTLs (0.017)
Sr	7.22 ± 4.86	1.96 ± 0.71	6.38 ± 2.21	4.27 ± 2.60	7.13	0.014	BTLs-GTBs (0.033)
Zn	53.70 ± 27.60	50.96 ± 13.12	52.02 ± 9.91	71.14 ± 10.26	3.33	0.073	–
Percentage contributions of the dialyzable fraction (mean values ± standard deviations)							
Al	1.19 ± 0.17	1.18 ± 0.23	5.02 ± 0.68	4.28 ± 1.36	50.18	0.000	BTBs-GTBs (0.000), BTBs-GTLs (0.000), BTLs-GTBs (0.000), BTLs-GTLs (0.000)
Ba	26.76 ± 1.78	33.04 ± 3.67	35.14 ± 3.74	48.46 ± 2.08	91.53	0.000	BTBs-BTLs (0.004), BTBs-GTBs (0.000), BTBs-GTLs (0.000), BTLs-GTLs (0.000), GTBs-GTLs (0.000)
Ca	41.94 ± 3.68	39.38 ± 6.84	47.68 ± 6.94	44.90 ± 2.76	1.64	0.251	BTLs-GTBs (0.027)
Cu	29.54 ± 3.91	20.70 ± 1.72	26.80 ± 4.50	20.76 ± 2.18	8.30	0.007	BTBs-BTLs (0.000), BTBs-GTLs (0.001), BTLs-GTBs (0.010), GTBs-GTLs (0.010)
Fe	9.40 ± 4.26	8.78 ± 0.96	12.00 ± 1.66	11.82 ± 2.73	4.91	0.032	–
Mg	39.64 ± 2.03	40.20 ± 2.00	43.88 ± 2.39	43.16 ± 2.25	4.05	0.045	BTBs-GTBs (0.007), BTBs-GTLs (0.021), BTLs-GTBs (0.016), BTLs-GTLs (0.047)
Mn	32.40 ± 1.41	33.40 ± 3.96	27.04 ± 5.50	32.88 ± 4.58	1.44	0.305	BTLs-GTBs (0.028)
Ni	21.12 ± 2.85	34.32 ± 8.05	17.44 ± 6.04	11.41 ± 2.63	16.17	0.001	BTBs-BTLs (0.001), BTBs-GTLs (0.012), BTLs-GTBs (0.000), BTLs-GTLs (0.000)
Sr	26.34 ± 3.48	22.12 ± 6.69	24.14 ± 2.49	48.92 ± 2.56	82.87	0.000	BTBs-GTLs (0.000), BTLs-GTLs (0.000), GTBs-GTLs (0.000)
Zn	41.26 ± 1.12	35.02 ± 2.72	33.90 ± 3.45	29.78 ± 2.84	26.96	0.000	BTBs-BTLs (0.002), BTBs-GTBs (0.000), BTBs-GTLs (0.000), BTLs-GTLs (0.007), GTBs-GTLs (0.027)

^a^The Welch test used to calculate values of the *F*-test (*α* = 0.05)

^b^Statistically significant differences between mean concentrations found using the Fisher least significant difference (LSD) test; *p* values given in brackets

Respective *F*-values and test significance (*p* values) showed that there were significant differences between these four groups of tea due to contributions of the bioaccessible fraction. In total, 31 differences were found between BTBs-BTLs (for Ba, Cu, Ni, Zn), BTBs-GTBs (for Al, Ba, Mg, Zn), BTBs-GTLs (for Al, Ba, Cu, Mg, Ni, Sr, Zn), BTLs-GTBs (for Al, Ca, Cu, Mg, Mn, Ni, Zn), BTLs-GTLs (for Al, Ba, Mg, Ni, Sr), and GTBs-GTLs (for Ba, Cu, Sr, Zn). Metals that distinguished mentioned groups of tea the most, in reference to contributions of the dialyzable fraction of their infusions, were Ba (5 differences out of 6 possible), Zn (5), Al (4), Cu (4), Mg (4), Ni (4), and Sr (3). Metals that did not differentiate analyzed teas or made it only to a small degree were Ca, Fe, and Mn.

Moreover, the number of differences between four groups of teas due to total concentrations of elements in infusions and concentrations of these metals in dialyzable and non-dialyzable fractions separated from infusions was very similar, i.e., 16 (infusions), 17 (the dialyzable fraction), and 16 (the non-dialyzable fraction). Infusions of analyzed BTBs, BTLs, GTBs, and GTLs were mostly differentiated due to concentrations of Al, Ba, Cu, and Ni. For these metals, the number of contrasts between four groups of teas was the highest. Metals such as Ca, Mg, Fe, and Zn were unfortunately established not to differentiate analyzed teas at all. The highest difference between total concentrations of studied metals was noted for Al and Fe. Accordingly, concentrations of Al in the dialyzable fraction determined in infusions of BTs and GTs were by 83 (BTBs)–90 (BTLs) and 20 (GTBs)–22 (GTLs) times lower, respectively, than total concentrations of this metal in infusions themselves. This pointed out that Al was strongly bound to the matrix of BTs, which is generally complicated, contains more complex condenses polyphenols as compared to the matrix of GTs [38, 39], and enables to readily complex Al (III) ions. Concentrations of Fe in the dialyzable fraction separated from infusions of BTs and GTs were also lower than total concentrations of this metal quantified in infusions themselves but to a lower degree, i.e., 11-fold for BTBs and BTLs, and 8.5 and 8.8 times for GTBs and GTLs, respectively. This data pointed out that Fe was also bound more strongly by the matrix of BTs than this of GTs due to obvious differences in composition of both types of tea [38, 39]. Anyway, this comparison strictly showed that GTs were a wealthier source of Al and Fe ready for the uptake by the body in the gastrointestinal track. In case of other metals, their concentrations in the dialyzable fraction were lower than their total concentrations determined in infusions but differences between BTs and GTs were less pronounced, i.e., × 3.0 (BTBs), × 3.7 (BTLs), × 2.9 (GTBs), and × 2.1 (GTLs) for Ba; × 2.3 (BTBs), × 2.5 (BTLs), × 2.1 (GTBs), and × 2.2 (GTLs) for Ca; × 3.4 (BTBs), × 4.9 (BTLs), × 3.8 (GTBs), and × 4.9 (GTLs) for Cu; × 2.5 (BTBs and BTLs), and × 2.3 (GTBs and GTLs) for Ca; × 2.9 (BTBs), × 3.1 (BTLs), × 3.1 (GTBs), and × 3.9 (GTLs) for Mn; × 3.8 (BTBs), × 4.3 (BTLs), × 2.0 (GTBs), and × 4.1 (GTLs) for Sr; and × 2.4 (BTBs), × 2.9 (BTLs), × 2.9 (GTBs), and × 3.4 (GTLs) for Zn. Behavior of Ni was different than for all other metals. The mean concentration of this metal in the dialyzable fraction of infusions of GTs was lower by 5.5- (GTBs) and 8.6-fold (GTLs) than its mean total concentration in infusions themselves. In case of BTs, the difference between concentrations in the dialyzable fraction distinguished in infusions and infusions themselves was twice lower, i.e., just × 2.9 (BTBs) and × 4.8 (BTLs).

**Fig. 2:**
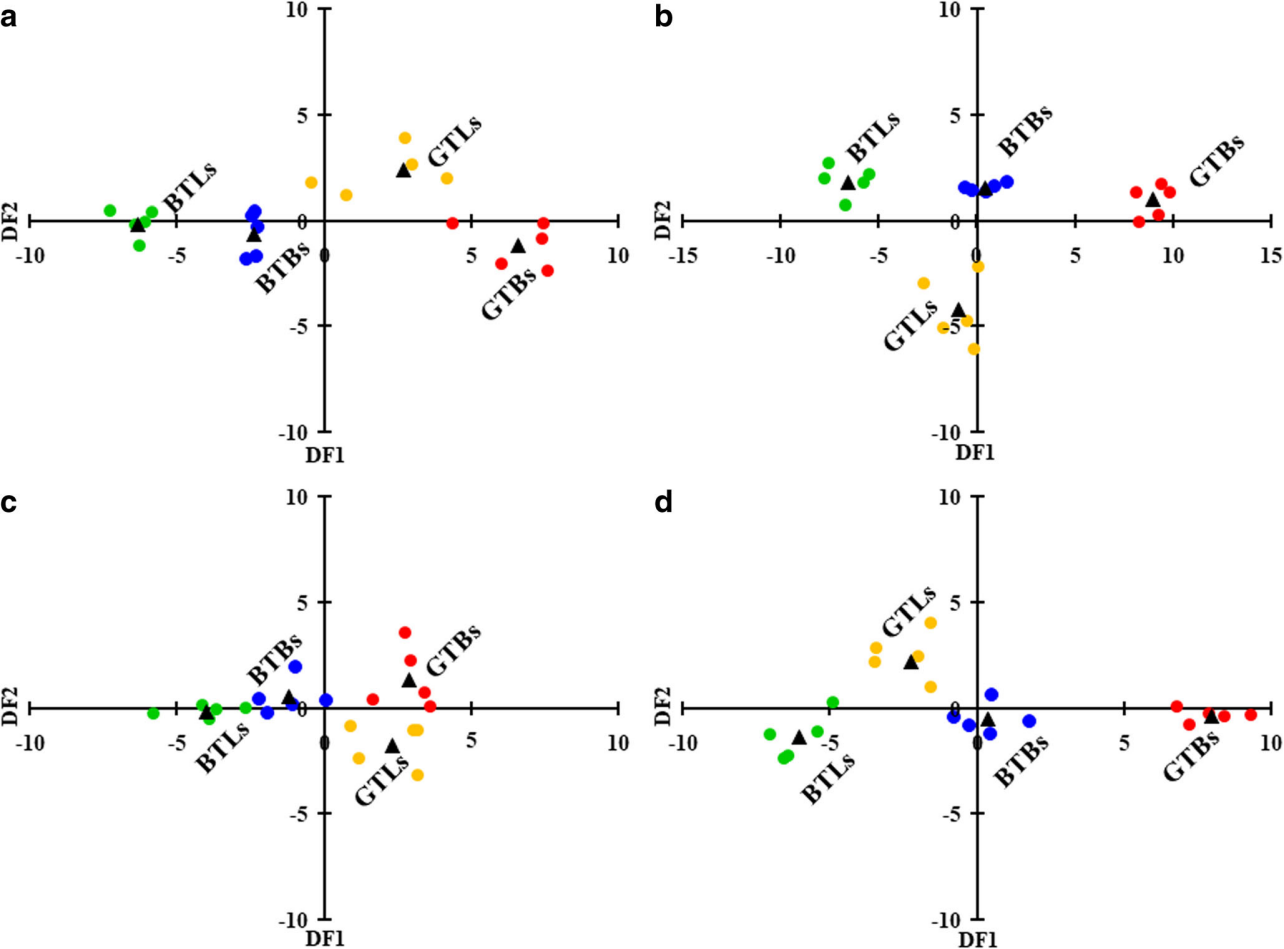
Two-dimensional scatter plots of two first discriminant functions (DF1 versus DF2) based on data matrices for all variables, i.e., total concentrations of metals determined in infusions of analyzed teas (**a**), and concentrations of metals in the dialyzable fraction separated from these infusions (**b**), or variables selected by the backward algorithm, i.e., total concentrations of Ba, Mg, Mn, Sr, and Zn in infusions of analyzed teas (**c**), and concentrations of Al, Ba, Ca, Cu, Mn, Ni, and Sr in the dialyzable fraction separated from these infusions. BTBs Bagged black teas (blue circles). BTLs Leaf black teas (green circles). GTBs Bagged green teas (red circles). GTLs Leaf green teas (yellow circles). Centroids (black triangles)

In addition, LDA was used to investigate possible classification of analyzed teas, i.e., BTBs, BTLs, GTBs, and GTLs. Results of this analysis are graphically presented in Fig. 2. Slightly better discrimination of four analyzed teas was obtained when concentrations of metals (all—Fig. 2b, or selected by the backward algorithm—Fig. 2d) determined in the dialyzable fraction separated from infusions of teas were used. In this case, the two first discriminant functions (DF1 and DF2) explained 97.5% (conditions I) and 97.9% (conditions II) of total variance. With respect to their classification models, LDA differentiated and correctly classified 100% of analyzed teas (20).

## Conclusion

The present study reports for the first time fully validated procedure for preparing sample of tea infusions after in vitro GID for the determination of 10 metals by spectrometric methods. Acidification with HNO_3_ to concentrations of 0.25 mol L^−1^ of the dialyzable fraction of infusions of BT and GT has been shown to be a reliable sample preparation procedure prior to determination of Al, Ba, Ca, Cu, Fe, Mg, Mn, Ni, Sr, and Zn by FAAS and ICP OES. This sample treatment significantly reduces time of analysis, requires minimal amounts of reagents, eliminates possible sample contamination, and demonstrates very good analytical performance. Therefore, it can successfully be used as a very good alternative to time-consuming, laborious, and inconvenient wet digestion procedures. Results of undertaken in vitro GID of infusions of BTs and GTs indicated that bioaccessibility of all determined metals from infusions of teas was lower than 50%. The most bioaccessible metal from infusions of BTs and GTs was Ca (mean contribution of the bioaccessible fraction of 40.7% and 46.3%, respectively), while the lowest bioaccessible was Al (mean contribution of the bioaccessible fraction of 1.2% and 4.6%, respectively). It was established that daily drinking of four cups of teas may cover RDIs of Ca, Cu, Fe, Mg, and Zn to a small degree (less than 2%). Only in case of Mn, it may contribute even up to 20% (infusions of BTs) and 33% (infusions of GTs) of the RDI for this metal. Additionally, based on PTWI values for Al and Ni, it was demonstrated that these metals, although present in infusions of analyzed BTs and GTs, may have rather negligible effects on health of tea consumers.

Multivariate analysis of data obtained for infusions of BTBs, BTLs, GTBs, and GTLs showed clearly that investigated teas were mostly differentiated due to concentrations of Al, Ba, Cu, and Ni.
